# Synthesis and characterization of Cu(II)-pyrazole complexes for possible anticancer agents; conformational studies as well as compatible *in-silico* and *in-vitro* assays

**DOI:** 10.1016/j.heliyon.2021.e08485

**Published:** 2021-11-26

**Authors:** Enas Aljuhani, Meshari M. Aljohani, Amerah Alsoliemy, Reem Shah, Hana M. Abumelha, Fawaz A. Saad, Aisha Hossan, Zehbah A. Al-Ahmed, Ahmed Alharbi, Nashwa M. El-Metwaly

**Affiliations:** a Department of Chemistry, Faculty of Applied Science, Umm Al-Qura University, Makkah, Saudi Arabia; b Department of Chemistry, Faculty of Science, Tabuk University, Tabuk 71491, Saudi Arabia; c Department of Chemistry, Faculty of Science, Princess Nourah Bint Abdulrahman University, Riyadh, Saudi Arabia; d Chemistry Department, Faculty of Science, King Khalid University, Abha, Saudi Arabia; e College of Art and Science, Dhahran Aljounb, King Khalid University, Saudi Arabia; f Department of Chemistry, Faculty of Science, Mansoura University, Mansoura, Egypt

**Keywords:** Cu(II) complexes, DFT, EPR, Anticancer agent, MDE-docking

## Abstract

New pyrazole derivatives were prepared and used to synthesize new bioactive agents from Cu(II) complexes that have OSN donors. Analytical and spectral (IR, UV-Vis, MS, ^1^H NMR, ESR & XRD) instruments characterized these complexes as well as their corresponding ligands. The bonding mode has been modified from ligand to ligand and the molar ratio for isolated complexes has also varied (1:1/1:2, M:L). The geometry of isolated complexes was commonly proposed, based on electronic transitions and ESR spectral-parameters. Via computational approaches, these structures were optimized using standard programs (Gaussian 09 & HyperChem 8.1) under the required basis set. Consequently, important physical characteristics have been obtained after finishing the optimization process. Inhibition behavior of all new synthesizes was studied by MOE module as *in-silico* approach which conducted versus the crystal structure of NUDT5 protein (6gru) of breast cancer cells. The interaction features summarized from docking processes, reveal effective inhibition validity for new Cu(II) complexes versus breast cancer cells. This according to scoring energy values and the stability of docking complexes in true interaction path (bond length ≤3.5 Å) particularly with Cu(II)-L^3^ and Cu(II)-L^4^ complexes. This reflects the possibility of successful behavior during practical application through *in-vitro* assay that intended in this study. Finally, the degree of toxicity of such new compounds to the breast cancer cell line was determined by *in-vitro* screening. To judge perfectly on their toxicity, *in-vitro* screening must compared to positive control as Doxorubicin (reference drug). IC_50_ values were calculated and represent Cu(II) complexes as outstanding cytotoxic agents which revealed superiority on the reference drug itself.

## 1 Introduction

A very dangerous tumor that destroys many women around the world is breast cancer [1]. While most traditional chemotherapies tend to be effective at the outset, they do not fully estrange the malignant clone. The real challenge is the changes in cancer cells that make them resistant to drugs [2, 3]. Recently, scientists are paying great attention to transition metal ion complexes derived from heterocyclic ligands due to their pharmacological and analytical uses [4]. Transition metal complexes with nitrogen- based ligands have attracted great interest, probably due to their significant intercalation with DNA bases [5]. Furthermore, Cu(II) ion has effective biological role with living systems as essential catalytic element [6]. Bioactive azo-dye ligand derived from pyrazole derivative was used to synthesize metal ion complexes which exhibited high antimicrobial activity [7].

Mononuclear Fe(II) complexes were obtained from 3,5-dimethyl-1-(2′-pyridyl)-pyrazole and the magnetic moment value was estimated at different temperature to investigate the relation in between [7]. In recent times, numerous reports have described anticancer vitality of copper complexes derived from many classes of nitrogen donors involving thiosemicarbazone, benzimidazole and pyridyl chelating ligands [8]. Among them, complexes containing pyrazole-based ligands have been reported as excellent antitumor agents which comparable with cisplatin drug [9]. Co(II), Ni(II) and Cu(II)-pyrazole complexes were prepared and characterized [10]. Additionally, other Cu(II)- pyrazole complexes displayed promising analytical and pharmacological applications [11].

Proceeding from previous literature outcomes and in continuation for our work [11], we planned to synthesize new bio-active copper complexes from pyrazole-based ligands. The new complexes were elucidated by available techniques to establish their chemical formulae. To obtain extensive physical properties for new compounds, intensive conformational analyses were performed. DFT method was applied *via* Gaussian 09 program to simulate x-ray single crystal analysis which we couldn't obtain. Furthermore, MOE-docking module was used to investigate the interaction behavior of tested compounds against breast cancer cell proteins to evaluate what happened inside the infected cell after treatment by a potential cytotoxic drugs as the Cu(II) complexes. Finally and according to *in-silico* data, *in-vitro* assay was carried out on new Cu(II) complexes against breast cancer cell line (MCF-7) to determine the extent of practical effectiveness and compared the results with that obtained from computational approach (*in-silico*).

## 2 Methods

All the chemicals or reagents utilized in this research were BDH and purchased from Merck and Sigma & Aldrich. The solvents implemented as ethanol, Dimethyl sulfoxide (DMSO) and Dimethyl formamide (DMF) were also spectroscopic and used without pretreatment. MCF-7 cell line was collected from American Type Culture Set Tissue Culture Unite, USA (ATCC).

### 2.1 Synthesis

#### 2.1.1 Synthesis of 4-phenylthiocarbamoyl-2-pyrazolein-5-one derivatives 3, 4 and 5(L^3–5^)

**Figure 1:**
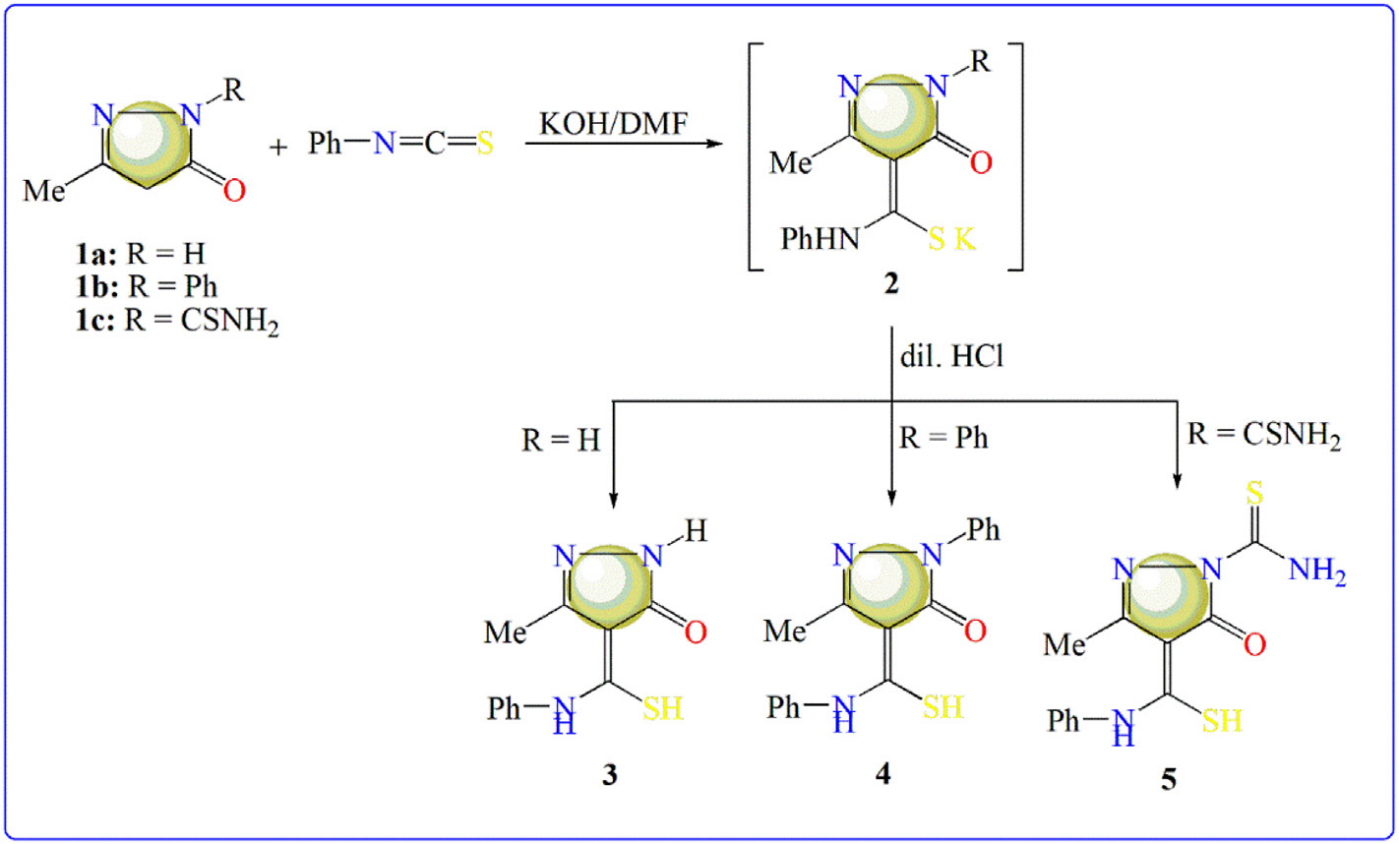
Synthesis protocol of pyrazinone derivatives.

To a cold suspension of finely divided KOH (0.01 mol, 0.56 g) in 20 mL DMF, pyrazolein-5-one derivative 1a, 1b and/or 1c (0.01 mol) was added and followed by phenyl isothiocyanate (0.01 mol, 1.20 ml). The mixture was stirred (at 25 °C) overnight, poured into ice-cold water and then neutralized by HCl (diluted). The solid obtained was collected by filtration and recrystallized from ethyl alcohol to afford corresponding 4-phenylthiocarbamoyl-2-pyrazolein-5-ones **3, 4** and **5**, respectively (Figure 1). We previously synthesized these derivatives to be used for complexation with VO(II) ion to prepare complexes for biological purpose, also [11].

3-Methyl-4-phenylthiocarbamoyl-2-pyrazolein-5-one (**3**) is a white solid; yield 58% and m.p. = 168–170 °C. IR spectrum (KBr) (Figure S1) exhibited bands at 3311 and 1643 cm^−1^ attribute to ʋ(N-H) and ʋ (C=O) vibrations, respectively. Also, ^1^H NMR spectrum (DMSO-*d*_6_, Figure S1) exhibited the following signals; δ/ppm = 2.22 (s, 3H, CH_3_), 6.73–7.22 (m, 5H, Ar-H), 9.21 (s, 1H, NH), 10.38 (s, 1H, NH). The mass spectrum (Figure S1) displayed molecular ion peak at m/z = 235.15(I = 0.2%), which corresponds to M^+^+ 2 with presence of sulfur isotope.

3-Methyl-1-phenyl-4-phenylthiocarbamoyl-2-pyrazolein-5-one (**4**) is a yellow solid; yield 58% and m.p. = 184–185 °C. IR spectrum (KBr) exhibited bands at 3099 and 1614 cm^−1^ attribute to ʋ(N-H) and ʋ(C=O) vibrations, respectively. Also, ^1^H NMR spectrum (DMSO-*d*_6_) displayed the following signals; δ/ppm = 2.34 (s, 3H, CH_3_), 7.13–7.76 (m, 10H, Ar-H), 10.15 (s, 1H, NH).

3-Methyl-4-phenylthiocarbamoyl-1-thiocarbamoyl-2-pyrazolein-5-one (5) is a yellow solid; yield 74% and m.p. = 114–115 °C (Lit. m.p. [12] 110–111 °C). IR spectrum (KBr) displayed the following bands; 3318, 3204, 3138 cm^−1^ which attributed to ʋ(NH_2_) and ʋ(NH) vibrations, while the band at 1654 cm^−1^ belongs to ʋ(C=O) vibration.

#### 2.1.2 Synthesis of Cu(II)-pyrazole complexes

**Figure 2:**
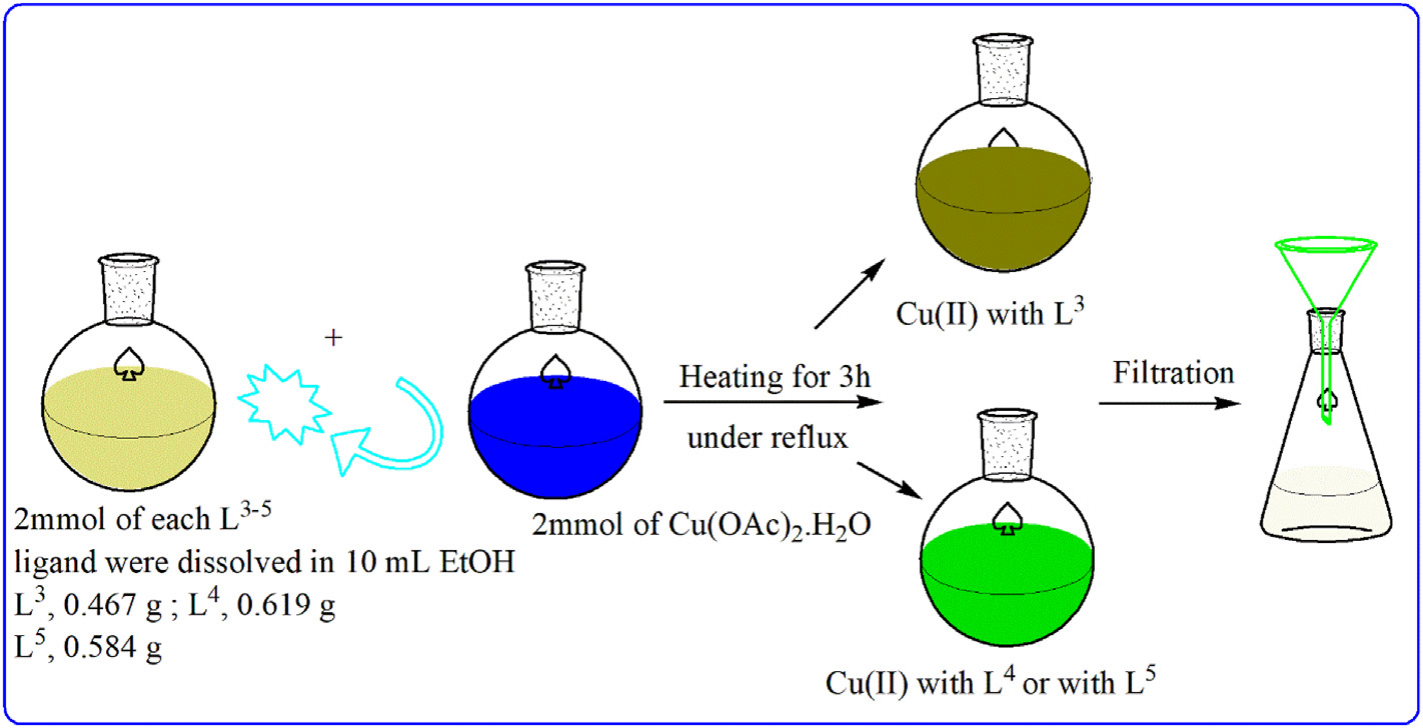
Synthesis outlines for Cu(II)- L^3**–**5^ complexes.

Each Cu(II) complex was prepared from Cu(OAc)_2_.H_2_O (2 mmol, 0.399 g) that dissolved in 5 mL bi-distilled H_2_O and then added to ethanolic solution of pyrazole derivative (2 mmol). Each reaction mixture was heated under reflux up to 3 h till the colored solid was appeared and kept to settle and then filtered off, washed by EtOH and dried over CaCl_2_ in closed desiccators. Notice; the synthesis process was schematically displayed for simplicity (Figure 2).

### 2.2 Analytical tools

Perkin-Elmer 2400 CHN Elemental Analyzer was used to extract the percentages of elements. Furthermore, the copper content of each complex was assessed using a complexometric titration technique, and the sulfur percentage was quantified gravimetrically using BaCl_2_ [13]. JENWAY model 4070 Conductance Bridge was used to acquire the conductance readings (in DMSO). The KBr-IR and NMR spectra were read using a JASCO FT-IR-4100 spectrophotometer (400–4000 cm^−1^) and a Burker (500 MHz). UV_2_ Unicam UV/Vis spectrophotometer (in DMSO solvent) and Johnson Matthey Magnetic Susceptibility Balance at 25 °C were used to get the electronic spectra and magnetic susceptibility, respectively. At 70 eV, mass spectra were obtained using an AEIMS 30 mass spectrometer with a heating rate of 40 °C/min and a mass/charge scanning range of 50–1000. At 25 °C, EPR spectra were obtained using Jeol JES-RE1X EPR spectrometer and a DPPH standard (υ = 9.435 GHz). Shimadzu Thermogravimetric Analyzer was used to extract TGA/DTG curves (20–900 °C). Under nitrogen, the heating rate was 10 °C min^−1^. On an X-ray diffractometer (GNR, APD2000PRO, Italy) with a graphite monochromator, XRD patterns (10°<2θ <90° rang) were recorded. Using Cu/Kα1 radiation, the scanning rate is 0.03° min^−1^.

### 2.3 Theoretical approach

#### 2.3.1 Optimization process

The structures of all synthesizes were optimized using a standard program like Gaussian 09 [14]. Energy minimization in ethanol default solvent was carried out under double-zeta basis set with polarization (6-31G∗) by DFT/B3LYP method. The target configurations and computational files were collected (log, chk & fchk). In order to extract critical characteristics, all files were visualized on Gauss screen [15] according to numbering scheme. Using the following equations [16, 17] and based on HOMO & LUMO energy gaps, further parameters were calculated:

χ = −0.5 (E_LUMO_ + E_HOMO_); μ = −χ = 0.5 (E_LUMO_ + E_HOMO_); η = 0.5 (E_LUMO_ – E_HOMO_); S = −0.5 η; ω = μ^2^/2 η and σ = 1/η

#### 2.3.2 Molecular operating environmental-docking (MOE)

**Figure 3:**
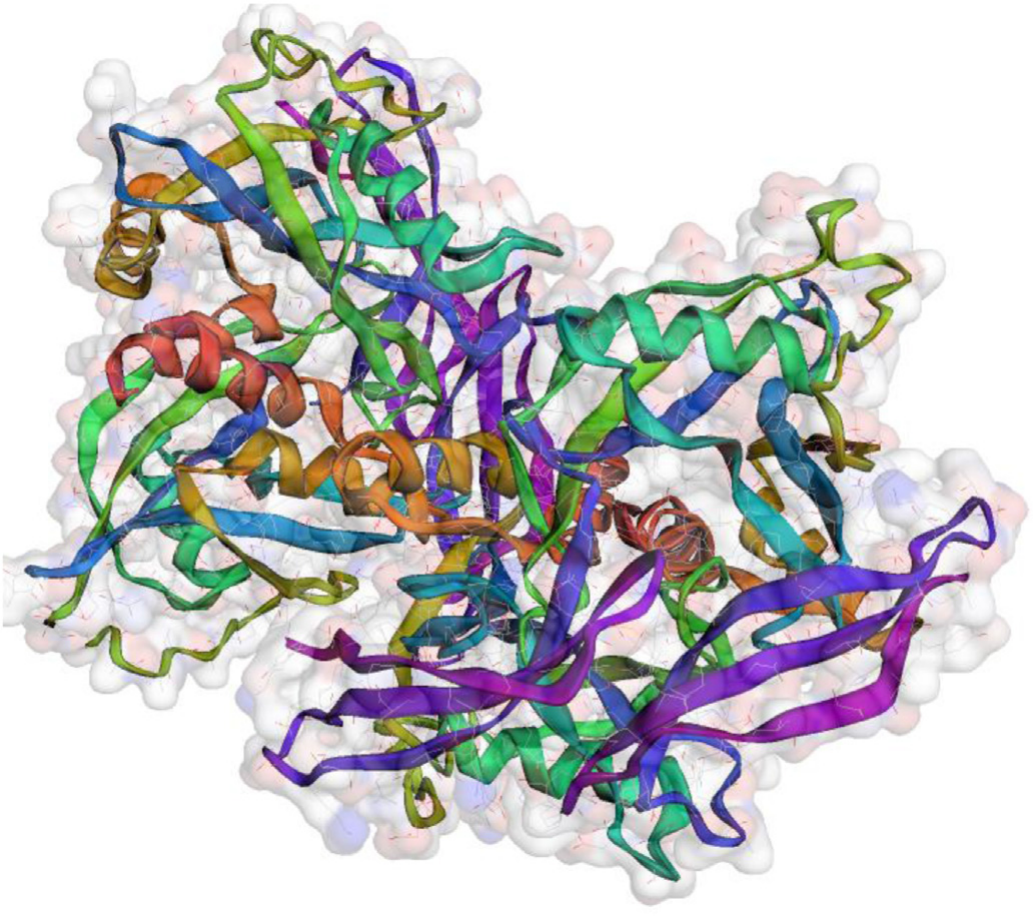
The characterization of crystal structure of NUDT5 protein of breast cancer (6gru).

Such *in-silico* technique was already approved by drug-designing industry and used in this study to simulate what happened inside infected cells after treatment with possible antitumor drugs (complexes). MOE-docking software (Vs. 2015), was used for this simulation to obtain the docking features towards the crystal structure of NUDT5 protein of breast cancer (6gru). To test inhibition activity, the interaction efficiency between suggested drug and cancer cell protein in its co-crystal form (as PDB file, Figure 3) was monitored. Each compound tested must be oriented *via* energy minimization during geometry optimization in the program.

After optimizing the structures, atomic charges were rendered followed by adjusting potential energy and other parameters were adapted by MMFF94x force field. Then save the oriented compound as MDB format in new database to be ready for the docking process. On the other hand, the protein used (6gru) must also be oriented by sequenced steps, starting by addition of hydrogen atoms over selected receptors. Then, the receptor types must be connected automatically and followed by fixing potential energy. After that, search on interaction sites and dummies over protein helix [18].

Consequently, select targeted MDB file and starting docking process which automatically ended after appearance of most suitable docking pose. Truly, the docking process is the mean of 30 poses and the time suitable for each process was varied from one to another. The poses were adjusted by London dG scoring function which improved twice-times by triangle Matcher. All interaction parameters, docking validity patterns and surface maps were exported. Docking score as well as the H-bond length (≤3.5 Å), are the significant indicators which used to evaluate and order the inhibition efficiency of the tested compounds.

### 2.4 In-vitro assay

In women, breast cancer is the second most common cause of cancer-related death. Depending on Society of American Cancer, thousands of breast cancer patients still need effective treatment all over the world. At varied doses (12.5, 25, 50, and 100 μM), the inhibitory effect of the new compounds on breast cancer cells was investigated. The reference used in this study was doxorubicin, which is one of the most effective anticancer drugs. The curve of breast cancer cell line (MCF7) survival, was achieved *via* plot between the relative viability (%) and concentration (μg/ml).

#### 2.4.1 Cytotoxicity

The toxic effect of the new Cu(II) complexes was evaluated by Mosmann's (MTT) (3-(4,5-dimethylthiazol-2-yl)-2,5-diphenyl tetrazolium bromide) test technique [19]. The Unite of Tissue Culture in Egyptian Organization of Vaccines & Biological Products provided us with MCF7 cancer cell lines from American Type Culture Collection. This assay was performed in specialized Laboratory, National Research Centre, and Cairo, Egypt. Sigma–Aldrich Chemical Company provided reagents and chemicals (St. Louis, MO, USA).

All of the following steps were conducted in a sterile environment with a biosafety class II level Laminar flow cabinet (Baker, SG403INT, Sanford, ME, USA). At 37 °C and 5% CO_2_, cells were suspended in DMEM medium for MCF7, 1% antibiotic-antimycotic mixture (10,000 U/ml Potassium Penicillin, 10,000 μg/mL Streptomycin Sulfate, and 25 μg/mL Amphotericin B), and 1% L-glutamine. Cells were batch grown for 10 days before being placed at a concentration of 10 × 10^3^ cells per well in freshly growth medium in 96-well microtiter plastic plates at 37 °C for 24 h in a water jacketed CO_2_ incubator (Sheldon, TC2323, Cornelius, OR, USA). The cells were kept either alone (negative control) or with varying quantities of sample to reach to a final concentration (100-50-25-12.5-6.25-3.125-0.78 and 1.56 ug/mL) after the media was aspirated and fresh medium (without serum) was applied. Medium was aspirated after 48 h of incubation, 40 ul MTT salt (2.5 μg/mL) was applied to each well, and the wells were kept for another additive 4 h at 37 °C under 5% CO_2_. 200 μL of 10% sodium dodecyl sulphate (SDS) in deionized water was added to each well and incubated overnight at 37 °C to end the process and dissolve the appeared crystals. A proven cytotoxic natural substance, 100 μg/mL, was utilized as a positive control, which gave 100% death under similar conditions [20, 21]. While, DMSO was used as a negative control based on using it to dissolve complexes to a final concentration on the cells of less than 0.2%. The absorbance was then measured at 595 nm with a reference wavelength of 620 nm using a microplate multi-well reader (Bio-Rad Laboratories Inc., model 3350, Hercules, California, USA). Statistical significance was estimated between samples and negative controls using the SPSS 11 program's independent t-test. According to known formula, the percentage of change in viability was computed [20].

## 3 Results and discussion

The molar ratio of isolated complexes was suggested to be 1Cu:1L except Cu(II)-L^3^ complex which has 1:2 M ratio according to analytical and physical characteristics (Table 1). Distinction ration emerged with the L^3^ ligand, due to its simple or slim structure which allows for the coordination of two ligand molecules without steric hinders. The apparent deep colors of complexes differed greatly from original ligands, due to high effect of charge transfer as well as d-d transitions.

**Table 1:** Physical and analytical properties of pyrazole derivatives (L^3–5^) and their complexes.

Compounds, (Empirical formula, Mwt. Calcd/Found)	Color	Elemental analysis (%) Calcd (Found)				
		C	H	N	S	Cu
1) L^3^(C_11_H_11_N_3_OS) (233.29/235.15)	White	56.63 (56.46)	4.75 (4.83)	18.01 (18.14)	13.74 (13.82)	–
2) [Cu(L^3^)_2_](OAc)_2_ (648.21/649.21)	Brown	48.18 (48.22)	4.35 (4.21)	12.96 (13.10)	9.89 (9.95)	9.80 (9.92)
3) L^4^(C_17_H_15_N_3_OS) (309.39)	Yellow	66.00 (66.12)	4.89 (4.78)	13.58 (13.45)	10.36 (10.66)	–
4) [Cu(OAc)_2_ (L^4^)](H_2_O) (509.03)	Bluish-green	49.55 (49.62)	4.55 (4.65)	8.25 (8.37)	6.30 (6.37)	12.48 (12.51)
5) L^5^(C_12_H_12_N_4_OS_2_) (292.05)	Yellow	49.29 (49.18)	4.14 (4.33)	19.16 (19.25)	21.93 (22.08)	–
6) [Cu(OAc) (L^5^) (H_2_O)](OAc) (H_2_O) (510.04)	Olive-green	37.68 (37.75)	4.35 (4.49)	10.98 (11.14)	12.57 (12.67)	12.46 (12.62)

### 3.1 IR-spectral data

IR spectral analysis (KBr-disc) was performed over 400–4000 cm^−1^ range for the ligands and their corresponding Cu(II) complexes (Figures S1 & S2), to extract significant vibrational bands (Table 2). Vibrations of υ(NH), υ(C=O), υ(C=N), υ(C-S) were the main bands specified in all pyrazoles, in addition to υ(C=S) and υ(NH_2_) bands in L^5^ derivative, only [22]. Comparing IR spectra of L^3^ and L^4^ ligands with their Cu(II) complexes, observable shift to lower wavenumber was recorded with υ(C=O) and υ(C-S) vibrations. This signifies the contribution of these groups in coordination *via* neutral bi-dentate mode of bonding. Furthermore, the conjugated anion (OAc^−^) was appeared either ionic or coordinating through bidentate mode with Cu(II)-L^3^ and Cu(II)-L^4^ complexes, respectively. While, the coordination of two ligands from L^3^ derivative in Cu(II)-L^3^ complex, eliminates the coordination of acetate group.

**Table 2:** Essential vibrational bands (cm^−1^) of pyrazole derivatives (L^3–5^) and their Cu(II) complexes.

Compounds	ν_(OH);_ ν_(NH)_	δ_(NH)_	ν_(C=O)_	ν_(C=N)_	ν_(C-S)_	δ_(OH)_	ν_as_, _s(OAc)_	δ_r_; δ_w(H2O)_	ν_M-O_	ν_M-S_
1) L^3^ (C_11_H_11_N_3_OS)	….; 3311	1492	1643	1550	689	–	–	–	–	–
2) [Cu(L^3^)_2_](OAc)_2_	….; 3440	1497	1596	1547	652	–	1454	–	557	505
3) L^4^ (C_17_H_15_N_3_OS)	….; 3099	1492	1614	1548	694	–	–	–	–	–
4) [Cu(OAc)_2_ (L^4^)](H_2_O)	B.c. at 3415	1497	1594	1543	651	1390	1453,1425	865; 620	575	508
	**ν**_**(NH2)**_**;ν**_**(NH)**_	**δ**_**NH2;NH**_			**ν**_**(C-S)**_**; ν**_**(C=S)**_					
5) L^5^ (C_12_H_12_N_4_OS_2_)	3318,3204; 3138	1593; 1492	1654	1532	698; 809	–	–	–	–	–
6) [Cu(OAc)(L^5^)(H_2_O)](OAc) (H_2_O)	B.c. at 3235	1581; 1516	1620	1581	650; 814	1319	1463,B at 1389	844; 757	582	503

B. c, broad centered.

During complexation, converged ionic and covalent properties of the acetate group results various interesting bonding modes. The differentiation between the two states, was basically depends on conductivity measurements (Table 3). While, the ionic and bidentate coordination mode could being discriminated from IR-spectra according to asymmetric and symmetric stretching vibrations (Table 2) [23]. The molar conductive values of complexes (Λ_m_, 1 mmol in DMSO) were varied and the conducting characteristic was predominant except for Cu(II)-L^4^ complex. Consequently, the conducting feature of Cu(II)-L^3^ and Cu(II)-L^5^ complexes, was suggested based on the obtained values (66.91 and 36.72 Λ_m_, Ohm^−1^ cm^2^ mol^−1^), respectively. Such values agree with presence of two acetate anions with Cu(II)-L^3^ complex, while only one with Cu(II)-L^4^ complex [24]. With respect to Cu(II)-L^5^ complex, the ligand binds as a neutral tridentate through ONS donors. This proposal is based on lower shifted appearance of υ(C=O), υ(NH_2_) and υ(C-S) vibrations, towards 1620, 3235 and 650 cm^−1^, respectively [25]. The solvent molecules were preliminary-proposed based on δ_r_(H_2_O) & δ_w_(H_2_O) vibrations, which strictly confirmed by thermal analysis (part 3.6). Consequently, υ(M-O) and υ(M-S) bands were assigned at lower wavenumber region in addition to υ(M-N) band in Cu(II)-L^5^ spectrum [25].

**Table 3:** Electronic transition bands (cm^−1^), magnetic moments and proposed geometries for Cu(II)-pyrazole complexes.

Complexes	Λ_m,_ Ohm^−1^ cm^2^ mol^−1^	μ_eff_ (BM)	Ligand field bands; assignments	charge transfer bands and intra-ligand transitions	Proposed geometry
1) [Cu(L^3^)_2_](OAc)_2_	66.91	1.74	20,833; ^2^B_1_g(d_z_^2^) → ^2^Eg(d_xz_, d_yz_) 9,524; ^2^B_1_g(d_z_^2^) → ^1^A_1_g(d_x_^2^_−y_^2^)	44,444; 26,316	Square-planer
2) [Cu(OAc)_2_(L^4^)](H_2_O)	4.67	1.90	10,000; ^2^Eg → ^2^T_2_g	44,843; 24,691	Distorted octahedral
3) [Cu(OAc)(L^5^)(H_2_O)](OAc)(H_2_O) 36.72		1.91	13,889; ^2^Eg → ^2^T_2_g	44,445; 27,027	Distorted octahedral

### 3.2 UV-Vis spectra and magnetic moments

Each complex scanned by UV-Vis radiation (150–1000 nm range, Figure S3) exhibited electronic transitions, which are essentially connected with the splitting in d-shell of metal ion as well as intra-ligand transitions. This study therefore assesses the degree of splitting corresponding to definite geometry. Intra-ligand transitions, charge transfer bands as well as *d-d* transitions were extracted and displayed (Table 3). Transitions of n → σ∗, π → π∗ and n → π∗ in the coordinating-ligands, were appeared in deep ultraviolet region [26]. While the ligand field transitions (LF) inside Cu(II)-L^3^ complex, two bands at 20,833 and 9,524 cm^−1^ were displayed. Respectively, these bands correspond to ^2^B_1_g(*d*_*z*_^*2*^) → ^2^Eg(*d*_*xz*_*,d*_*yz*_) and ^2^B_1_g(*d*_*z*_^*2*^) → ^1^A_1_g(*d*_*x*_^*2*^_−*y*_^*2*^) transitions in square-planer geometry.

**Figure 4:**
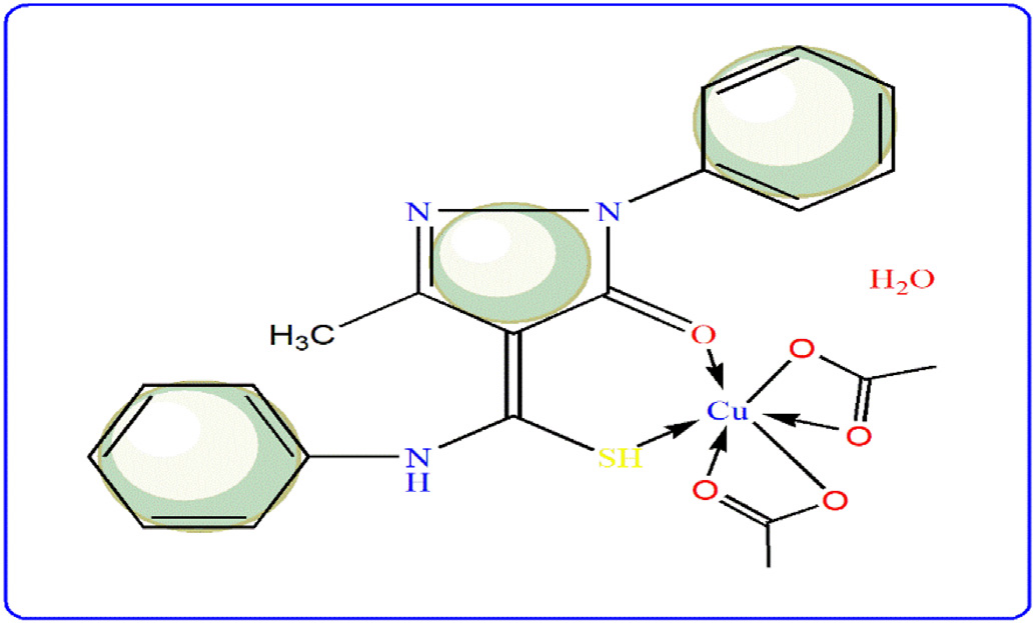
Structural form of Cu(II)-L^4^ complex.

While, UV-Vis spectra of Cu(II)-L^4^ and Cu(II)-L^5^ complexes, close to octahedral geometry (Figure 4), which must be distorted based on Jahn-Teller effect [27]. This geometry suggested depends on existence of only one band in each spectrum at 10,000 or 13,889 cm^−1^ which attributes to ^2^Eg → ^2^T_2_g, transition, respectively [27]. Finally, due to presence of orbital-orbital contribution, the measured magnetic moment values (μ_eff_, BM) were changed from spin-only moment values (1.73 BM) [28, 29, 30].

### 3.3 ESR spectra

Electron paramagnetic resonance is a significant tool that provides further validation of previously proposed structural forms (UV-Vis part). For instance, two complexes were chosen for this scanning at υ = 9.435 GHz (DPPH standard, at 25 °C) (Figure S4). Four hyperfine splitting lines (S = 1/2, I = 3/2), were expected for resolved microstates (m_l_ = 3/2, 1/2, −3/2 & −1/2) in the complexes, while the shortage of resolution may prohibit clear isolation. Hamiltonian g values (Table 4), were g_//_>g_⊥_>2.0023 agree with octahedral geometry according to ^2^A_1_g (d_X_^2^_−Y_^2^), as a ground term [31]. The g-values are strongly affected by spin-orbital coupling (λ = −828), which significantly changed after complexation. Exchange interaction parameter G was calculated and their values were extremely lower than the normal value (G = 4) [32]. This is due to presence of metal-metal interaction between neighboring molecules, which affecting on hyperfine splitting clarity. Also, tetrahedral distortion index (f = g_//_/A_//_), was calculated for such complexes. The values calculated (132.869 &135.154) are fairly close to that known for octahedral configuration [33].

**Table 4:** ESR spectral data for two selected complexes (A and p ×10^−4^).

Complex	g_//_	g_⊥_	g_o_	A_11_	f	A_⊥_	A_o_	G	p	k	α^2^	β^2^
1) [Cu(OAc)_2_(L^4^)](H_2_O)	2.1459	2.1022	2.1168	161.505	132.869	60.841	94.396	1.4374	285.597	0.0062	0.6750	0.3212
2) [Cu(OAc)(L^5^)(H_2_O)](OAc)(H_2_O)	2.1737	2.0990	2.1239	160.831	135.154	67.724	98.760	1.7725	287.083	0.0134	0.6996	0.5137

Moreover, isotropic and anisotropic parameters were also calculated for them by using the following relations; A_o_= (A_//_+2A_⊥_) ⁄ 3 **(1)** and g_o_ = (g_//_+2g_⊥_) ⁄ 3 **(2)** [34]. The values calculated (A_o_ & g_o_) are deviated much from free electron state, due to complexation and distortion. The influence of unpaired electron on inner atomic-core, was also calculated, as a Fermi-contact term (k). This parameter was estimated from this relation K = -(A_o_/p)-(g_e_ - g_o_) **(3)** and the values were normal. The in-plane σ-bonding (α^2^) and in-plane π-bonding (β^2^) values, were calculated by α^2^ = (A_//_/0.036)+(g_//_ − 2.0023)+3/7(g − 2.0023)+0.04 **(4)** and β^2^ = (g_//_ − 2.0023)E/(−8λ α^2^) **(5)** equations [35]. Their values point to covalent characteristic of new Cu-L bonds (near 0.5). The term of dipole was computed for the complexes through this relation P = 2 γCu β_o_ β_N_/(r−3) **(6),** where, γCu is the magnetic moment, β_N_ is the nuclear magneton, β_o_ is the Bohr magneton and r is the ionic radius [36]. Finally, these parameters appeared normal with that known for the geometries proposed with high covalence of Cu(II)-L bonds.

### 3.4 Mass spectral analysis

**Figure 5:**
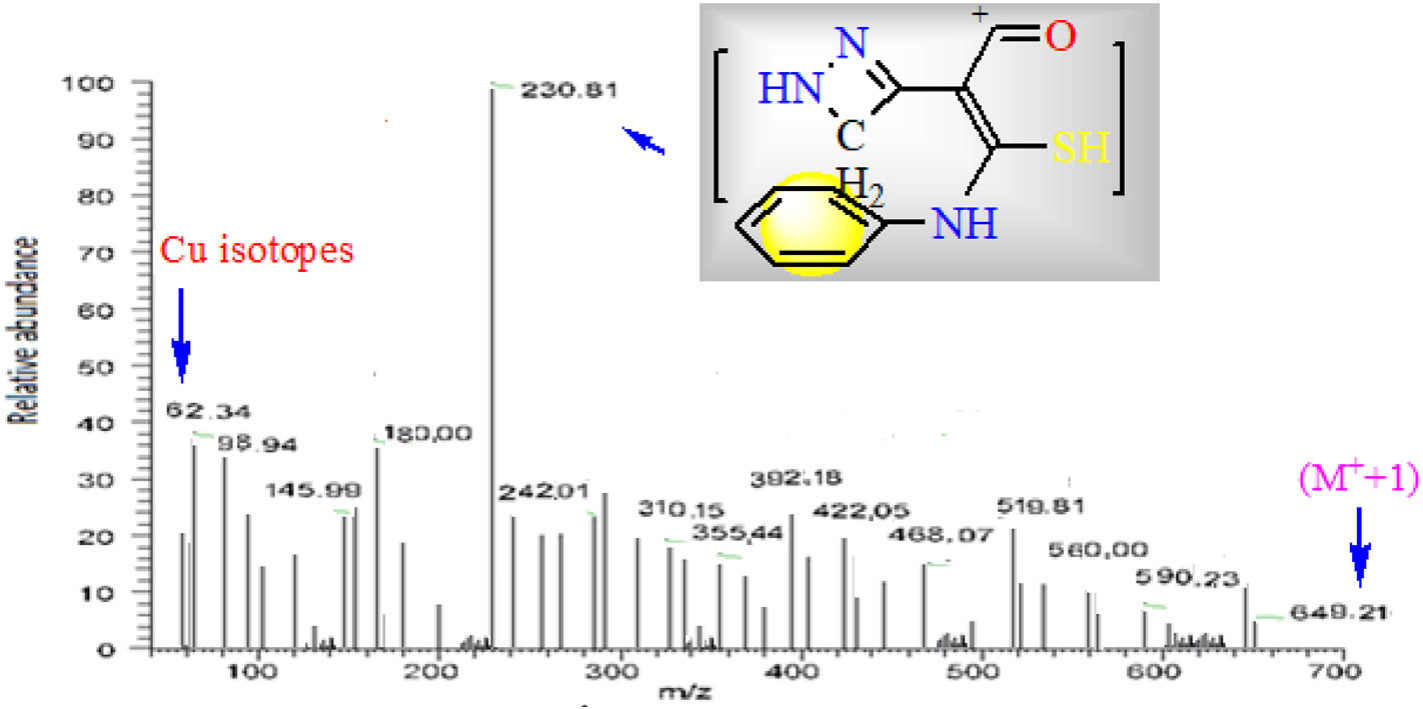
Mass spectrum of Cu(II)-L^3^ complex.

A chosen complex (for example) was analyzed by this effective analytical tool which used if we could not isolate a single-crystal from the tested compound. This scanning was conducted over a wide mass range (m/z = 50–1000) at 70 eV and under 40 °C/min heating rate. Fragmentation was done by electron bombardment after vaporizing the complex and leads to multiple ions which separated according to their m/z ratio. Therefore, the molecular ion peak was recorded firstly and then followed by multiple fragmentation peaks. Molecular ion peak was appeared at m/z = 649.21 and matched with M^+^ + 1 ion (I = 4%). Also, the expected fragment suitable to base peak was depicted on the figure (Figure 5). In addition to, the influential-copper isotopes peaks, were clearly appeared.

### 3.5 TGA study

Thermogravimetric analysis is a supporting method used for solid complexes to check the existence or absence of water molecules. This study was carried out for complexes over 20–800 °C range (Figure S5) at constant heating rate (10 °C min^−1^). Plausible degradation paths were aggregated (Table S1) to assert on some properties. Lower thermal stability was recorded with Cu(II)-L^4^ and Cu(II)-L^5^ complexes due to presence of crystal water molecules. All complexes were completely degraded over three to four stages till reach to 500–700 °C range. The residual portion was suggested to be CuO which contaminated by carbon atoms.

### 3.6 XRD patterns description

**Figure 6:**
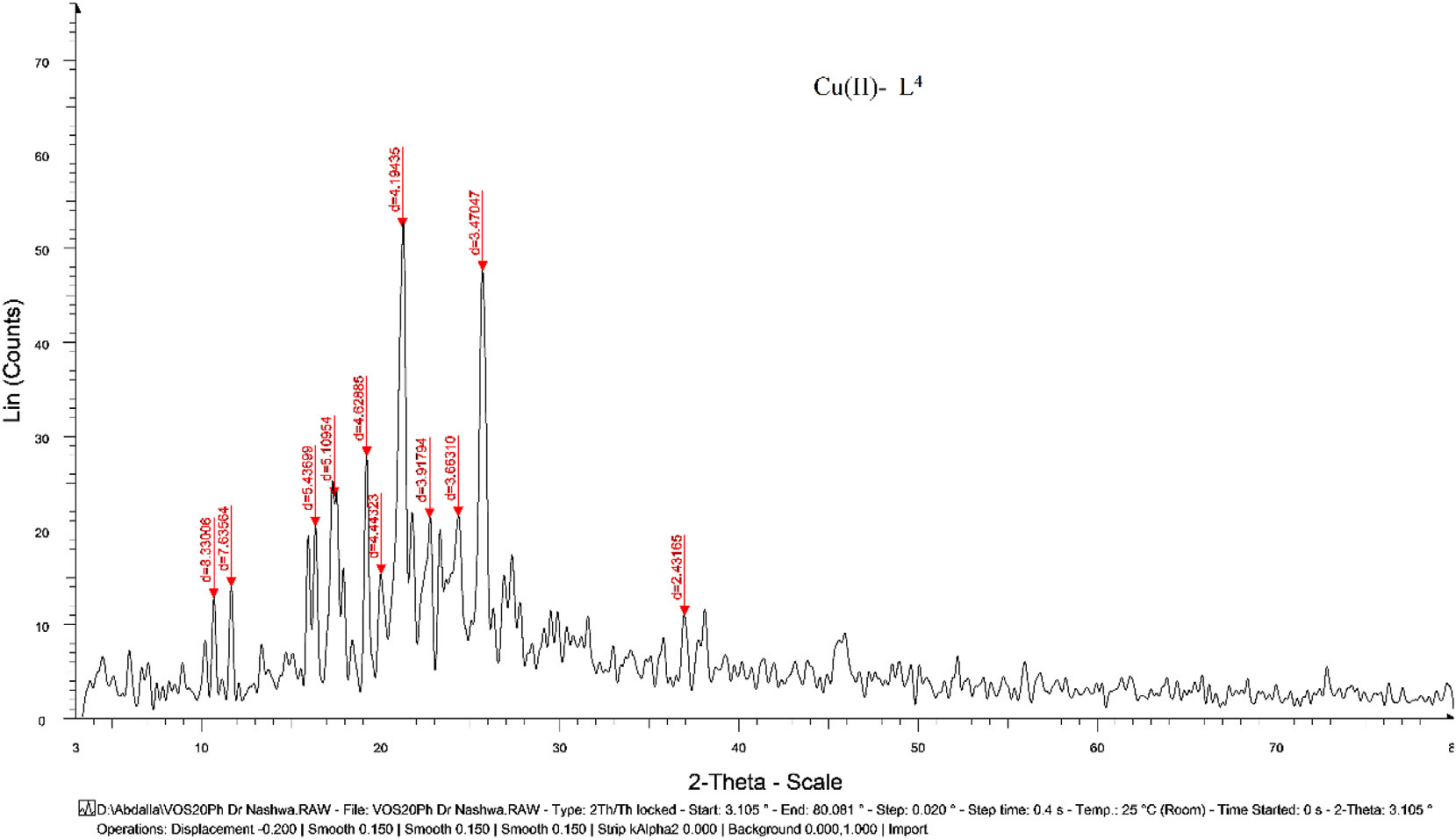
XRD pattern for the best nano-crystalline Cu(II)-L^4^ complex.

X-ray diffraction patterns was obtained by using Cu/Kα1 radiation-source to confirm the crystallinity, purity and lattice dynamics of solid compounds under 2θ = 10°<2θ < 80° range (Figures 6 & S6). The patterns reflect excellent crystallinity of Cu(II)-L^4^ complex, while the rest complexes were fairly amorphous [37]. Using FWHM method, crystallite-parameters were estimated and displayed (Table 5) to evaluate extensive properties. Particulate sizes, d_hkl_-spacing, relative intensity, crystal strain (ε) and dislocation density (δ), were the crystal-lattice parameters calculated by using high intense peak [38, 39]. The measured particle sizes were appeared in the nano-meter range, excellently. The lower crystal strain and dislocation values, indicate that the solids appeared having fairly perfect crystals .

**Table 5:** Crystallite parameters using FWHM method for fairly crystalline complexes.

Compounds	Size (Å)	2θ	Intensity	d-spacing (Å)	ε	δ (Å^−2^)	FWHM
1) [Cu(L^3^)_2_](OAc)_2_	2.5346	6.891	40.7	12.8173	0.6067	0.1557	0.5724
2) [Cu(OAc)_2_(L^4^)](H_2_O)	2.5955	21.165	52.3	4.1945	0.1940	0.1484	0.5676

### 3.7 Conformational study

Molecular modeling program as Gaussian 09 offers an interesting simulation for x-ray single crystal analysis which we couldn't easily obtain it due to failure to reach to a single crystal and lack of availability of the device. But the molecular modeling programs yield a highly credible data could confirm the formula of compounds suggested.

**Figure 7:**
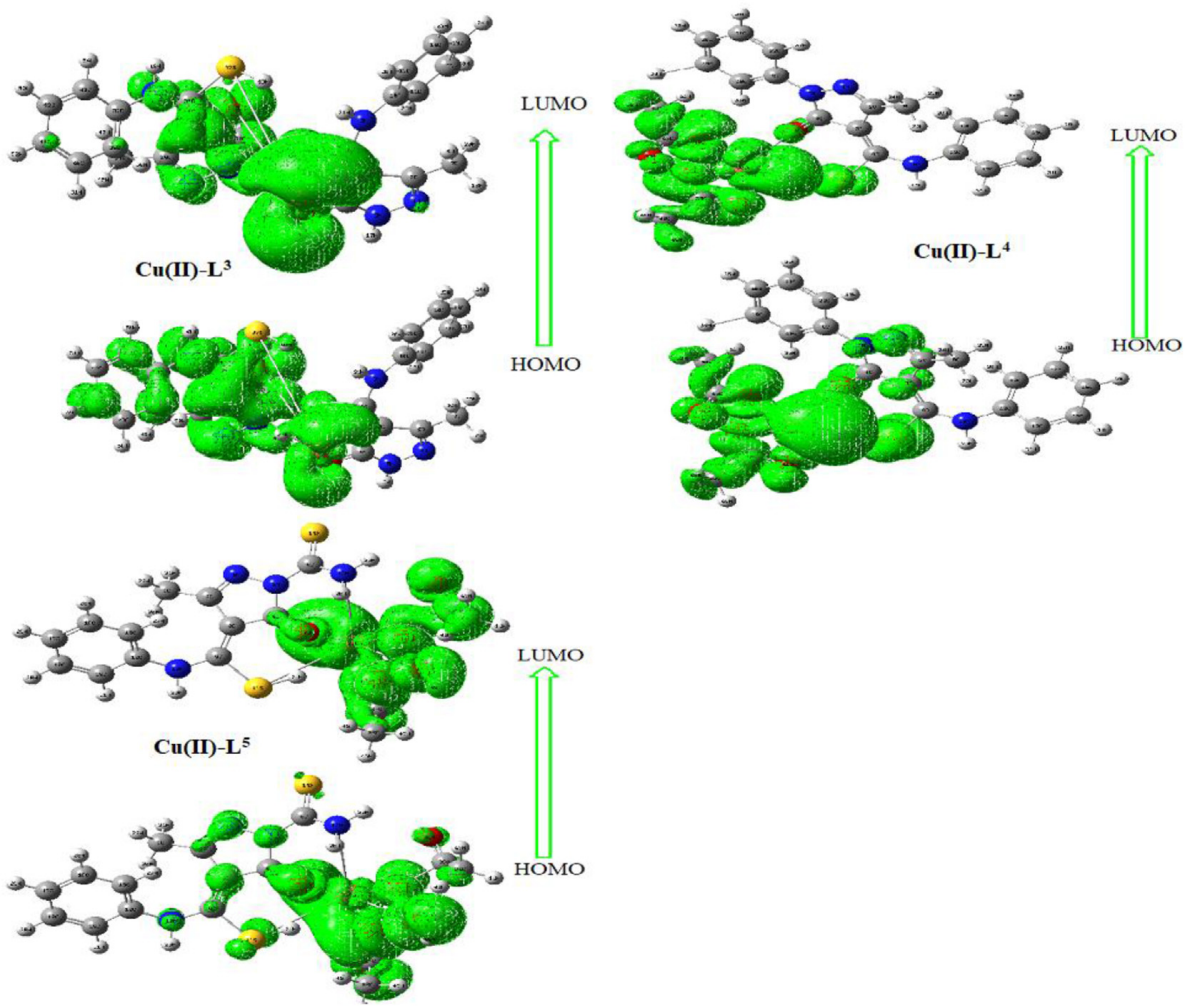
HOMO & LUMO images for Cu(II)-L^3^, Cu(II)-L^4^ and Cu(II)-L^5^ complexes.

The optimized structures of new synthesizes were achieved by using DFT/B3LYP method under most fitted basis set (6-31G∗) (Figures S7 & S8). Regarding pyrazole-based ligands (L^3–5^, Figure S7), the distribution of C-SH, C=O functional groups enhances their coordination with copper center as already proposed (from spectral analysis) beside NH_2_ group in L^5^ derivative. The charges of pyrazole derivatives (Figure S9) reveal the high negativity on centers selected for coordination as appeared in the figure. Frontier molecular orbitals (HOMO and LUMO), were extracted (Figures 7 & S10) from fchk files and appeared distributed along the whole ligands, while appeared concentrated around copper center in complexes.

Based on energy gaps between two molecular orbitals (ΔE = E_LUMO_-E_HOMO_), functional indexes were evaluated by known equations [15, 16]. Such indexes were electrophilicity (ω), global-hardness (η), global-softness (s), absolute-softness (ϭ), electronegativity (x) and chemical potential (μ). Furthermore, dipole moment (D) and formation energy (E, a. u.) values were extracted from log files and also exhibited (Table S2). Structural activity relationships can be summarized as follows;

I) For all complexes, significantly reduced energy gaps emerged, indicate the effect of metal atoms on electronic transition within the complex [40, 41].
II) Due to their effect on compound behaviour during biological application, enhanced electrophilicity and absolute-softness lead to biological dominance of complexes.
III) On the other side, hardness index as well as dipole moment values, maintain the reverse relationship with biological effectiveness.

Consequently, increasing the polarity over the molecule, decreases lipophilicity for the compound. So, the polar compound cannot miscible with lipid to interact with biological systems. Although, some organic derivatives have low dipole moments (L^3,4^), but their therapeutic role is not preferable than the complexes. This is due to the essential role of metal atom, which binds with the base pairs in amino acids of biological systems as proteins or enzymes [42, 43]. This interaction leads to blocking the active sites in the system and then inhibit its biological role.

#### 3.7.1 Electrostatic potential maps (MEP)

**Figure 8:**
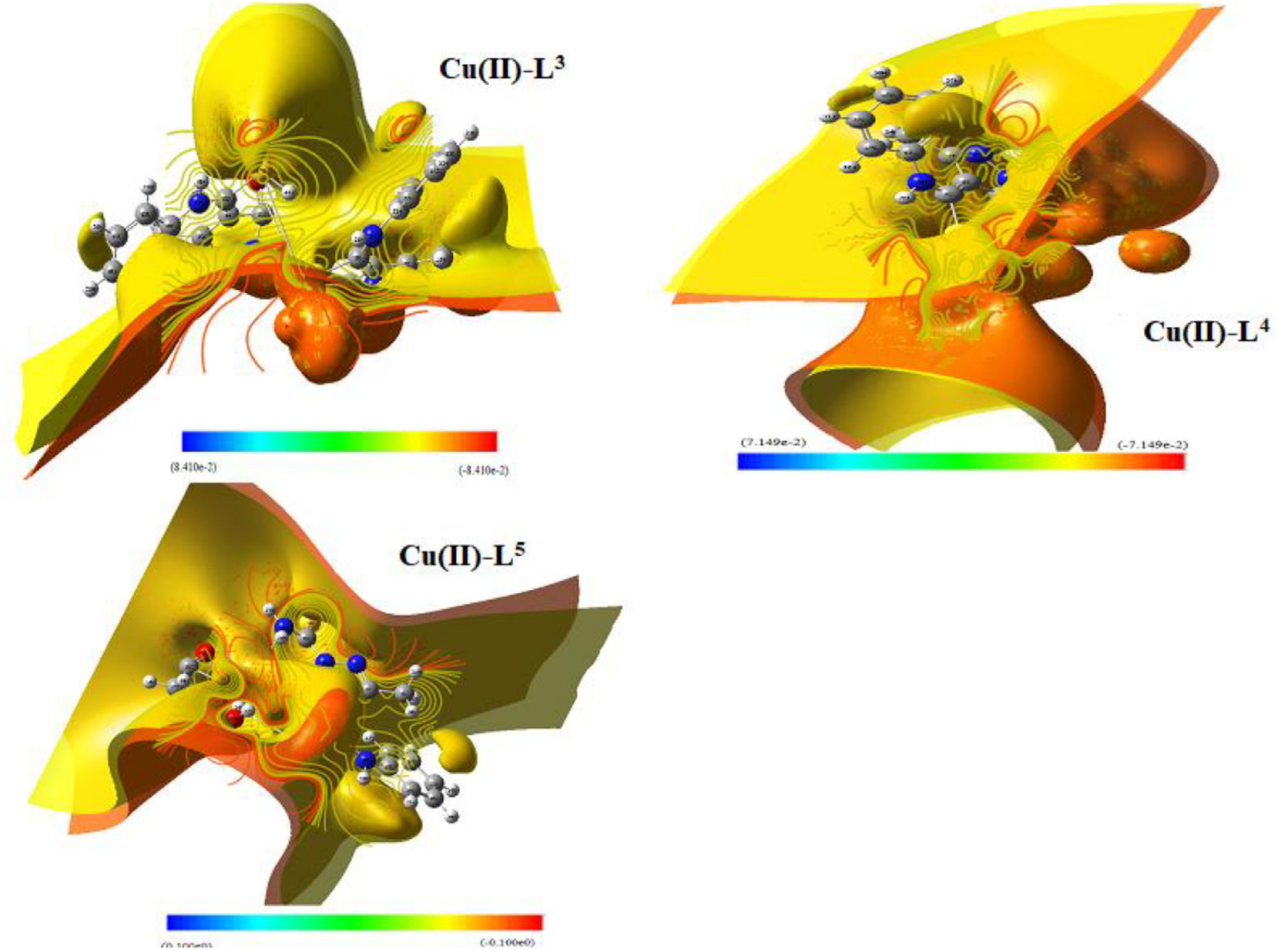
Iso-surface of ESP contour map of Cu(II)-L^3^, Cu(II)-L^4^ and Cu(II)-L^5^ complexes.

The maps of MEP and iso-surface with array-plot were constructed for all compounds *via* chk and fchk files on new cubic contour (Figures 8 & S11). Some features attached to compound reactivity are highlighted in these charts. By using red, blue and green colors, MEP maps (Figure S11) clearly distinguish the nucleophilic, electrophilic and zero potential areas. While, iso-surface with array-plot maps (Figure 8) exhibited observable broadness for inner core (yellow lines), while the outer core (red lines) seems reduced. This reflects the shortage of unsaturated boundary surrounds the complexes.

### 3.8 OSAR parameters

Quantitative Structural Activity Relationship (QSAR), was evaluated for all compounds after their optimization by using HyperChem (8.1) program. To optimize the structures, firstly add the H-atoms over the molecule, setup semi-empirical (AM1) which followed by setup Molecular Mechanics force field (MM^+^). The process proceeded without fixation for any other parameters under Polake-Ribiere conjugated gradient algorithm method [44]. The determined parameters have been aggregated (Table S3) and compared. In general, reactivity values suggest the dominance of complexes over the free ligands [45]. In accordance with Gaussian 09 findings, partition coefficient values (Log p) and polarizability index, which are inversely proportional to biological efficiency, showed general superiority of complexes, especially for Cu(II)-L^5^ [46].

### 3.9 MOE-docking analysis

**Figure 9:**
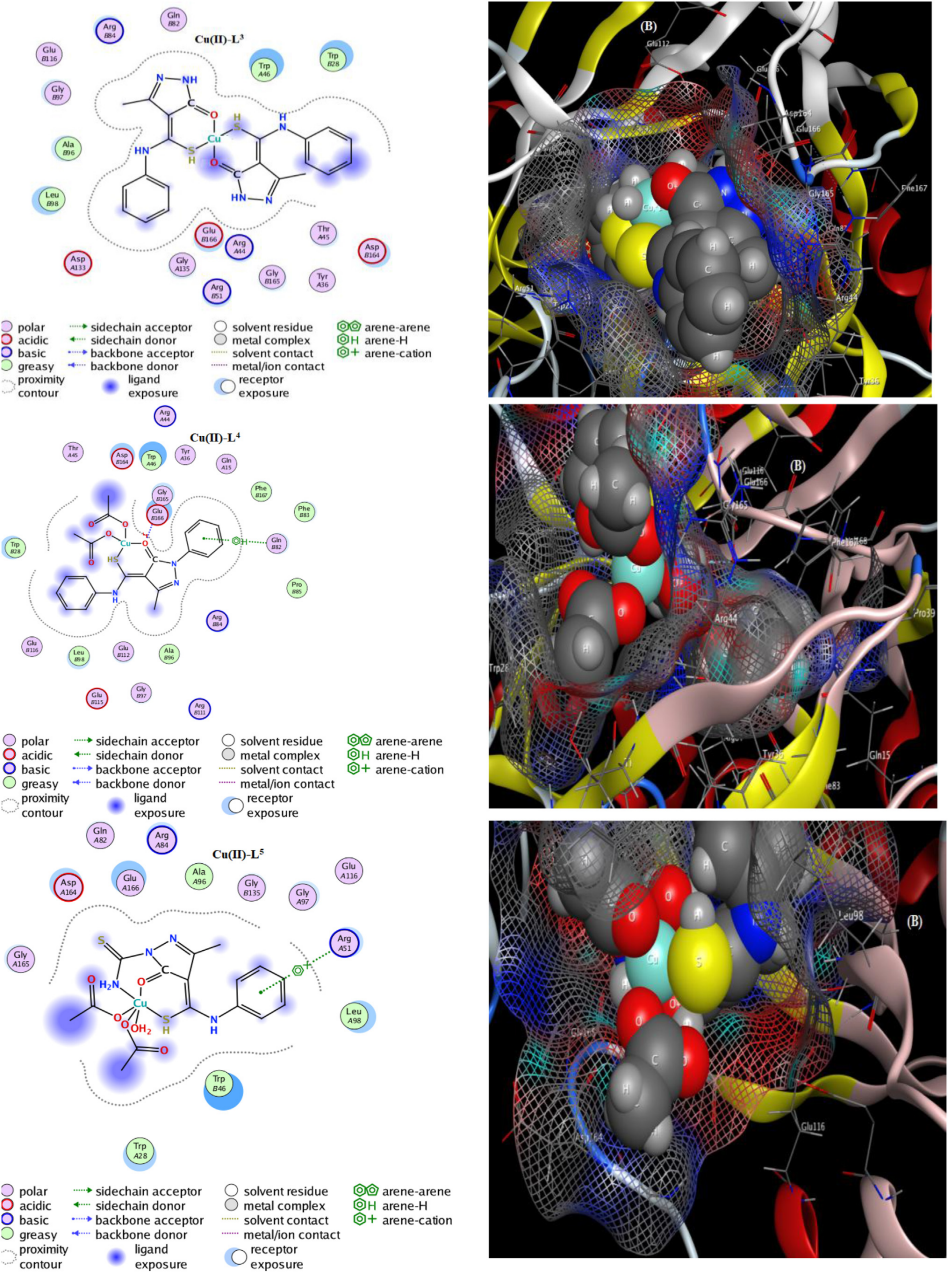
The docking validity (A) and interaction potential (B) maps of Cu(II)-L^3^, Cu(II)-L^4^ and Cu(II)-L^5^ complexes against 6gru protein.

In drug industry, *in-silico* study using MOE module was utilized for docking process between the suggested drug (tested compound) and infected cell protein to determine the extent of inhibition of designed drug. Here, we focused on testing the ligands and their Cu(II) complexes towards crystal structure of NUDT5 protein of breast cancer (6gru). Although, the indexes calculated formerly (parts 3.8 & 3.9) offer a well expectation for influential inhibition, but docking simulation is still the most effective method for deliberate evaluation. The data exported clarify without doubt the inhibition feature of the tested complexes towards breast cancer cell protein which exceeded than the free ligands [47]. All interaction features were obtained and displayed both in Table 6 and in Figures 9 & S12. Most of docking patterns are excreted from true docking paths based on the length of H-bonding (≤3.5 Å) formed between the compound and protein pockets [48]. According to scoring values displayed in Table 6, the docking poses of Cu(II)-L^5^-6gru, Cu(II)-L^4^-6gru and Cu(II)-L^3^-6gru may be the best inhibitors. But, the docking path within the Cu(II)-L^5^-6gru complex, is untrue due to unacceptable length for the H-bond formed (3.98 Å) which exceeds 3.5 Å. Also, the interaction in Cu(II)-L^3^-6gru docking pose are not known, all the parameters couldn't obtained. In addition, we aggregated other essential docking features from the poses obtained as follow;

1 The values of energy score were −6.974, −6.727, −6.604, −6.500, −5.498 and −5.258 for Cu(II)-L^5^, Cu(II)-L^3^, Cu(II)-L^4^, L^4^, L^5^ and L^3^, respectively. The complexes have high values which is a favorable feature.
2 The binding sites from the compounds were S10, N1, O7 and 6-ring which are the only sites interacted with residues through allosteric binding types of H-donor, H-acceptor and Pi-cation.
3 The interacting residues or dummies were GLY135 (B), GLU47(B), GLN82(A), PHE167(A) and ARG51(A)
4 With respect to docking poses of Cu(II)-L^5^-6gru and Cu(II)-L^4^-6gru, acidic receptors were contributed through sidechain acceptor or donor type, also basic receptors were contributed through backbone acceptor type. While, with respect to the docking pose of L^5^-6gru, the polar, nonpolar, basic and acidic receptors were contributed in bonding with different sites.
5 The ligand exposure surface is mostly reduced in all docking poses which reflect the degree of saturation for the compound surface with bonding which cannot exceeded. This is also observed from the broadness of proximity contour (dotted line surrounds the compound) [49].
6 Moreover, the content of energy inside the docking poses yielded are moderate from −0.7 to −4.1 kcal/mol (Table 6).
7 Docking proteins have a broad electrostatic surface, which is observed in most complexes, implying that they can easily penetrate protein grooves generated by helix degradation during contact.

**Table 6:** Docking parameters for all synthesizes against functional breast cancer protein (6gru).

Complex	Ligand	Receptor	Interaction	Distance (Å)	E (Kcal/mol)	S (energy score)
1) L^3^	S10 N1	O GLY 135 (B) N GLU 47 (B)	H-donor H-acceptor	3.25 3.56	−1.1–0.7	−5.258
2) Cu(II)-L^3^	–	–	–	–	–	−6.727
3) L^4^	–	–	–	–	–	−6.500
4) Cu(II)-L^4^	O7	O GLY 165 (B)	H-donor	2.94	−3.1	−6.604
5) L^5^	O7 S14 S14	NE2 GLN 82 (A) NE2 GLN 82 (A) N PHE 167 (A)	H-acceptor H-acceptor H-acceptor	2.98 3.25 3.48	−1.7 − 1.8 −4.1	−5.498
6) Cu(II)-L^5^	6-ring	NH1 ARG 51 (A)	Pi-cation	3.98	−0.7	−6.974

### 3.10 In-vitro anticancer screening

Cancer therapy is a significant obstacle faced by scientists involved in bio-active agent synthesis. We predicted the good inhibitory effect of such complexes from the background of copper complexes as well as from theoretical aspects estimated in this study. New Cu(II) complexes were screened *in-vitro* against breast cancer cell line (MCF-7). A series of concentrations (100, 50, 25, 12.5. 6.25, 3.125–0.78 and 1.56 μg/ml) from each complex was examined to calculate IC_50_ values and compared to Doxorubicin drug. The values estimated (Table 7), indicate cytotoxicity of Cu(II)-L^5^ and Cu(II)-L^3^ complexes, which superior the reference drug itself. Such complexes may be considered as anticancer agents which must be studied intensively from specialists.

**Table 7:** Cytotoxic data for all complexes against MCF-7 cell line.

Complexes	In vitro cytotoxicity IC_50_ (μg/ml)	Toxicity percentage (%)
Doxorubicin	26.1 ± 0.2	100
1) Cu(II)-L^3^	25.14 ± 1.7	103.82
2) Cu(II)-L^4^	30.06 ± 2.0	86.83
3) Cu(II)-L^5^	9.12 ± 0.7	286.18

## 4 Conclusion

The complexes of Novel Cu(II) were synthesized and thoroughly characterized. Molecular and structural formulae of new compounds are elucidated by spectral and analytic techniques. Conductivity measurements and TGA tests were used to validate the presence or absence, respectively, of acetate and water molecules in the coordination sphere. All compounds were geometrically optimized by using Gaussian 09 and HyperChem programs to extract significant physical characteristics. Using the MOE docking module, as *in-silico* method was applied to determine the inhibition grade of new compounds towards breast cancer cell protein (6gru) before *in-vitro* assay. The outputs predict that, the Cu(II) complexes will have promising antitumor efficiency. As a result, *in-vitro* assay was performed for Cu(II) complexes against breast cancer cell line to measure the extent of inhibition for such complexes. The toxicity of two copper complexes surpassed that of reference drug itself (Doxorubicin).

## Declarations

### Author contribution statement

Enas Aljuhani; Meshari M. Aljohani: Conceived and designed the experiments.

Amerah Alsoliemy; Reem Shah: Performed the experiments.

Nashwa M. El-Metwaly: Analyzed and interpreted the data; Wrote the paper.

Hana M. Abumelha; Fawaz A. Saad; Aisha Hossan: Contributed reagents, materials, analysis tools or data.

Zehba A. Al-Ahmed; Ahmed Elharbi: Conceived and designed the experiments; Contributed reagents, materials, analysis tools or data.

### Funding statement

This work was supported by the Deanship of Scientific Research at UmmAl-Qura University (Grant Code: 19-SCI-1-01-0039).

### Data availability statement

Data will be made available on request.

### Declaration of interests statement

The authors declare no conflict of interest.

### Additional information

No additional information is available for this paper.

## Appendix A Supplementary data

Supplementry final.docx Supplementry final.docx
