# Supplementary material for: Synthesis and characterization of Cu(II)-pyrazole complexes for possible anticancer agents; conformational studies as well as compatible in-silico and in-vitro assays
Source: Heliyon. 2021 Nov 26;7(11):e08485. doi: 10.1016/j.heliyon.2021.e08485 (PMC8639435; doi:10.1016/j.heliyon.2021.e08485)
Supplement: Supplementry final.docx [file mmc1.docx]

| L^3^ |  |
| --- | --- |
| L^3^ | 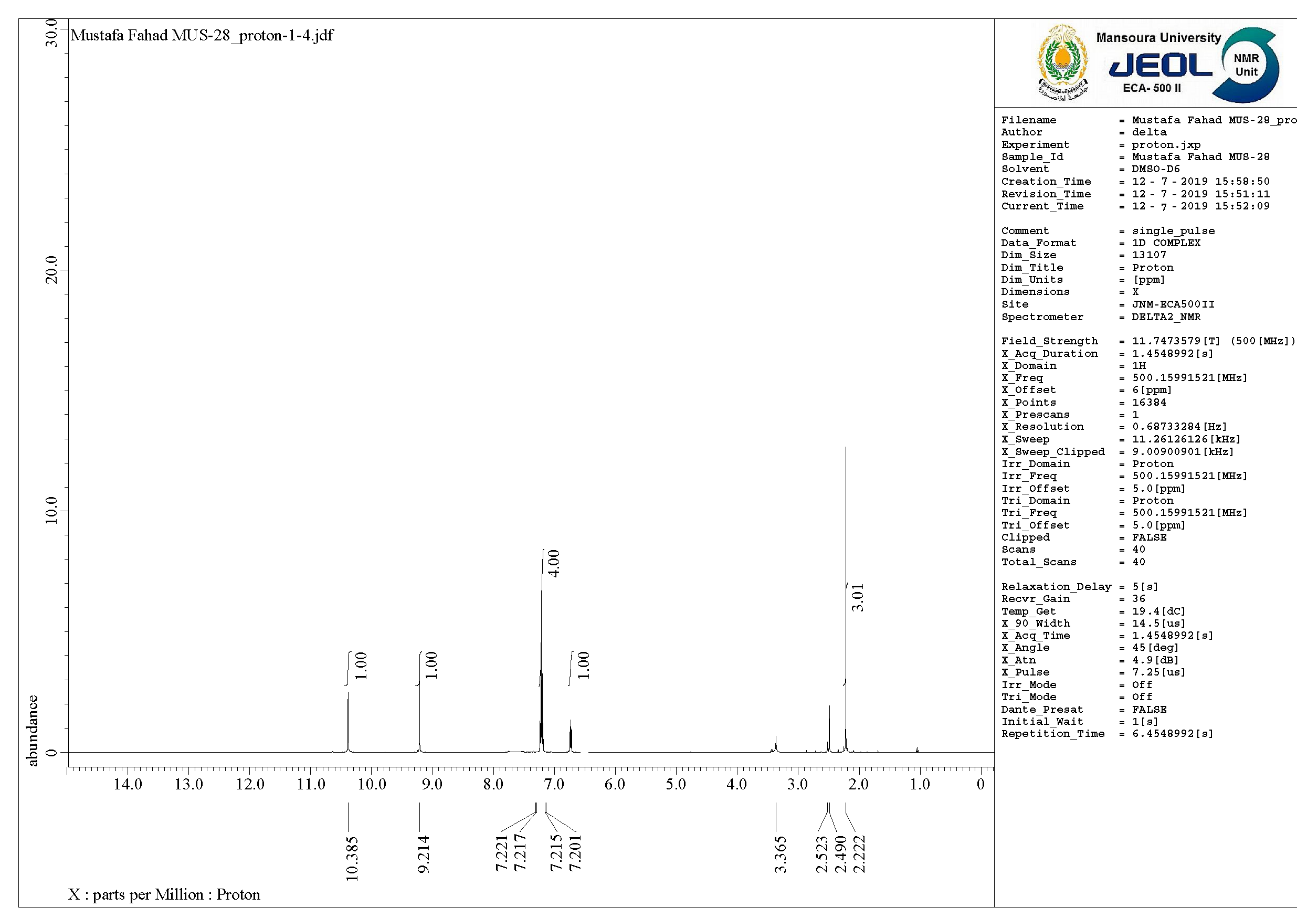 |
| L^4^ |  |
| L^4^ | 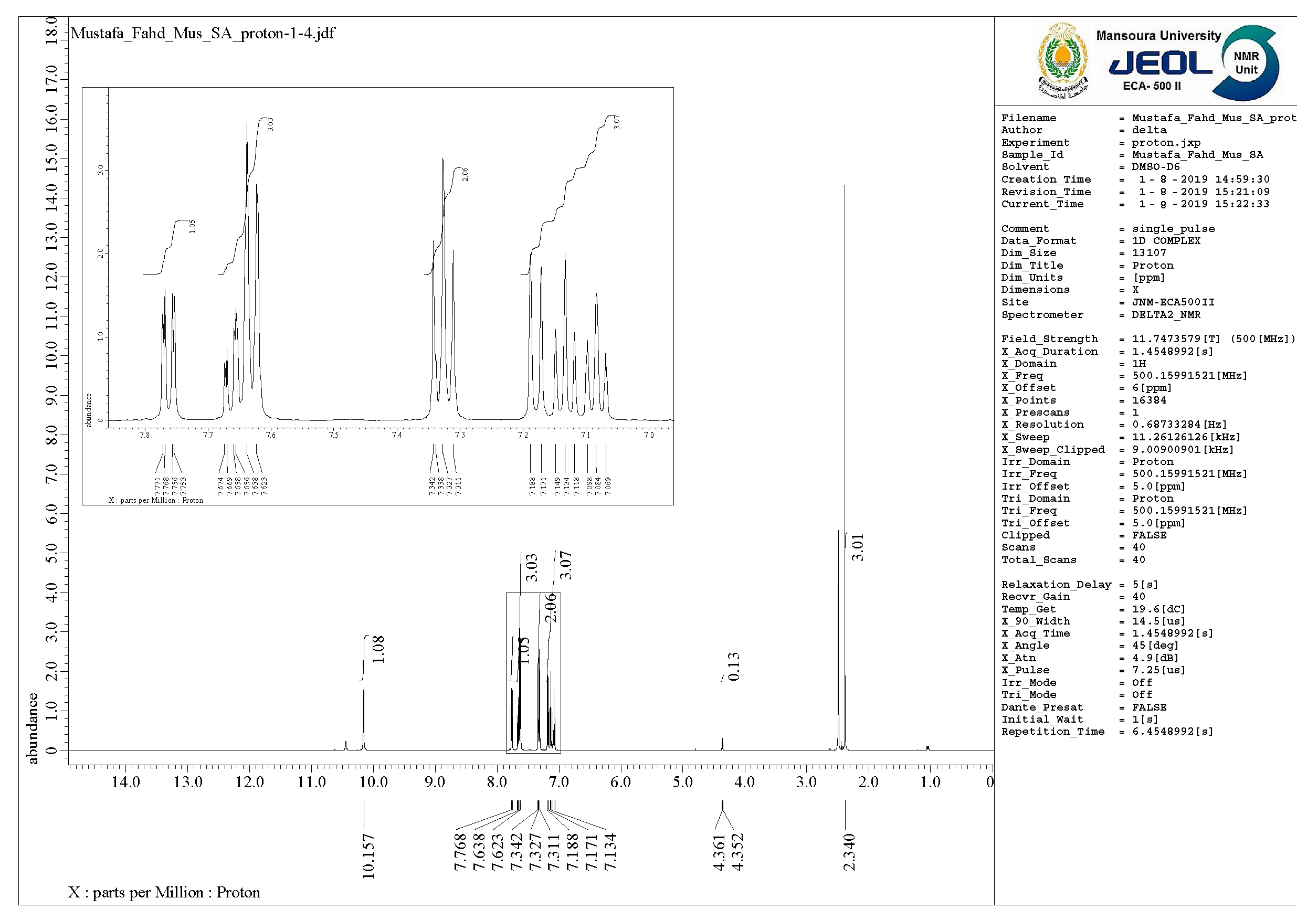 |
| L^5^ |  |
|  | 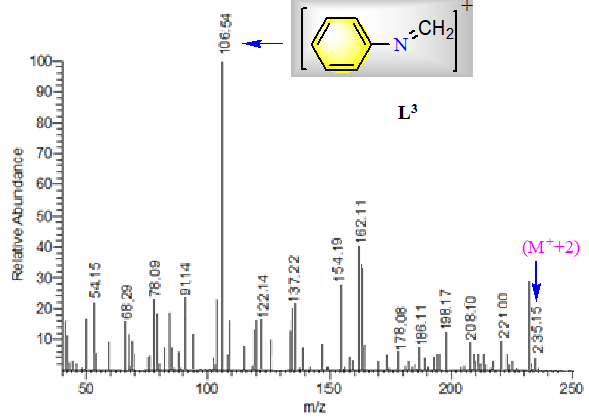 |
| **Figure S1** IR**,** ^1^H NMR & mass spectra of pyrazole derivatives (L^3-5^) | |

| Cu(II)-L^3^ |  | | |
| --- | --- | --- | --- |
| Cu(II)-L^4^ |  | | |
| Cu(II)-L^5^ |  | | |
| **Figure S2.** IR spectra of Cu(II)-L^3^ , Cu(II)-L^4^ and Cu(II)-L^5^ complexes | | | |
| 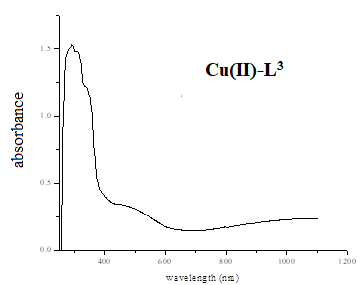 | | 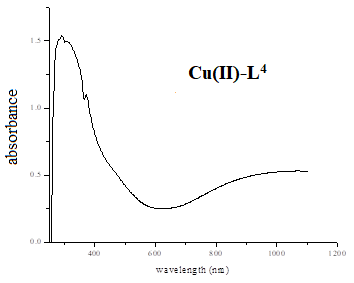 |  |
| 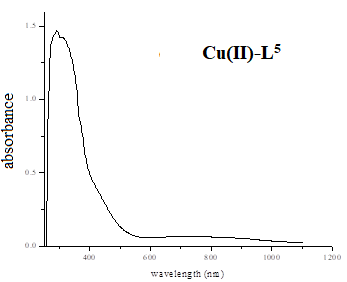 | |  |  |
| **Figure S3.** UV-Vis spectra (in DMSO) for Cu(II)-L^3^ , Cu(II)-L^4^ and Cu(II)-L^5^ complexes | | |  |

| 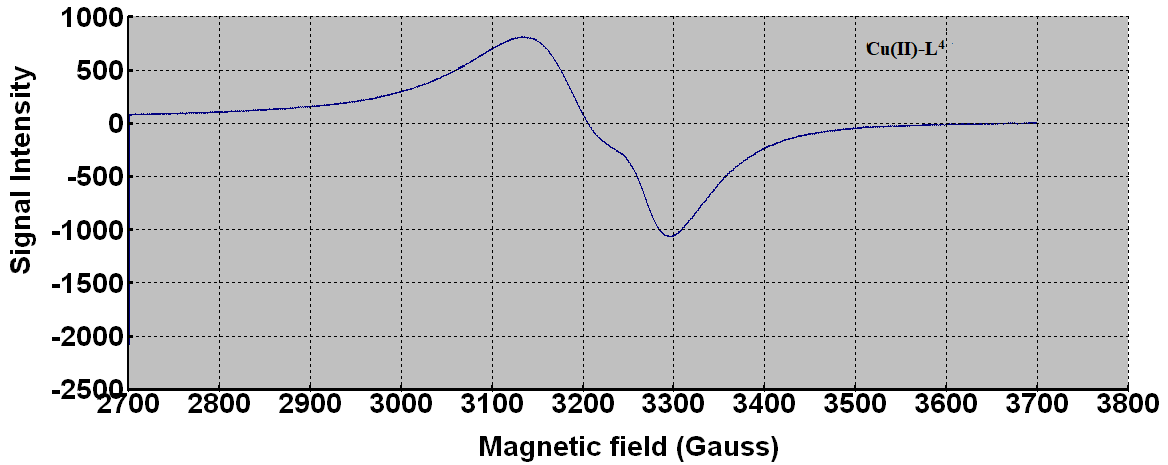 |
| --- |
| 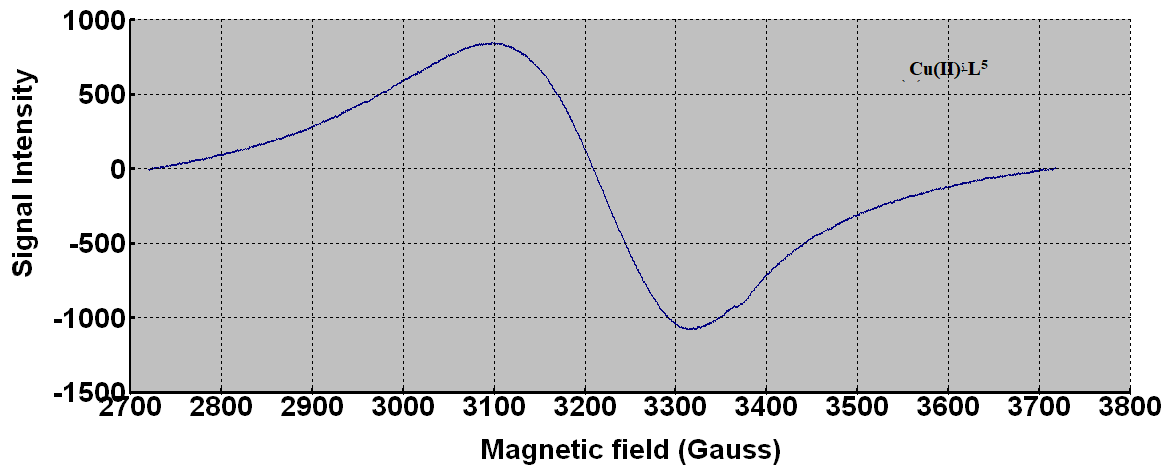 |
| **Figure S4.** ESR spectra for selected Cu(II)-L^4^ and Cu(II)-L^5^ complexes |

| 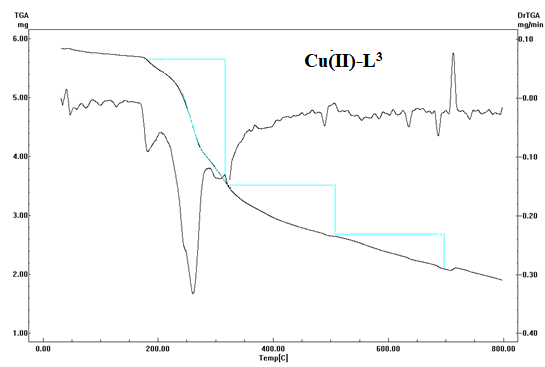 | | | | | |
| --- | --- | --- | --- | --- | --- |
| 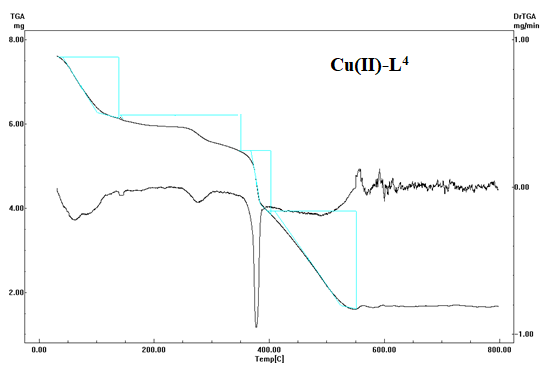 | | | | | |
| 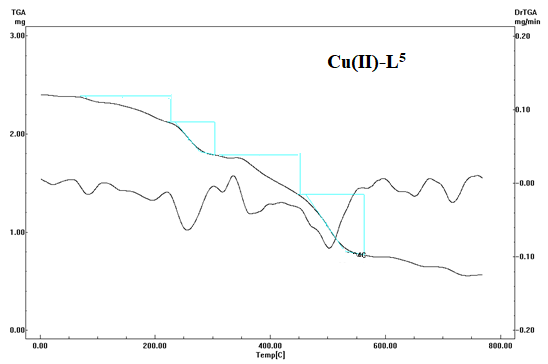 | | | | | |
| **Figure S5** TGA analysis curves for Cu(II)-L^3^ , Cu(II)-L^4^ and Cu(II)-L^5^ complexes | | | | | |
| \| 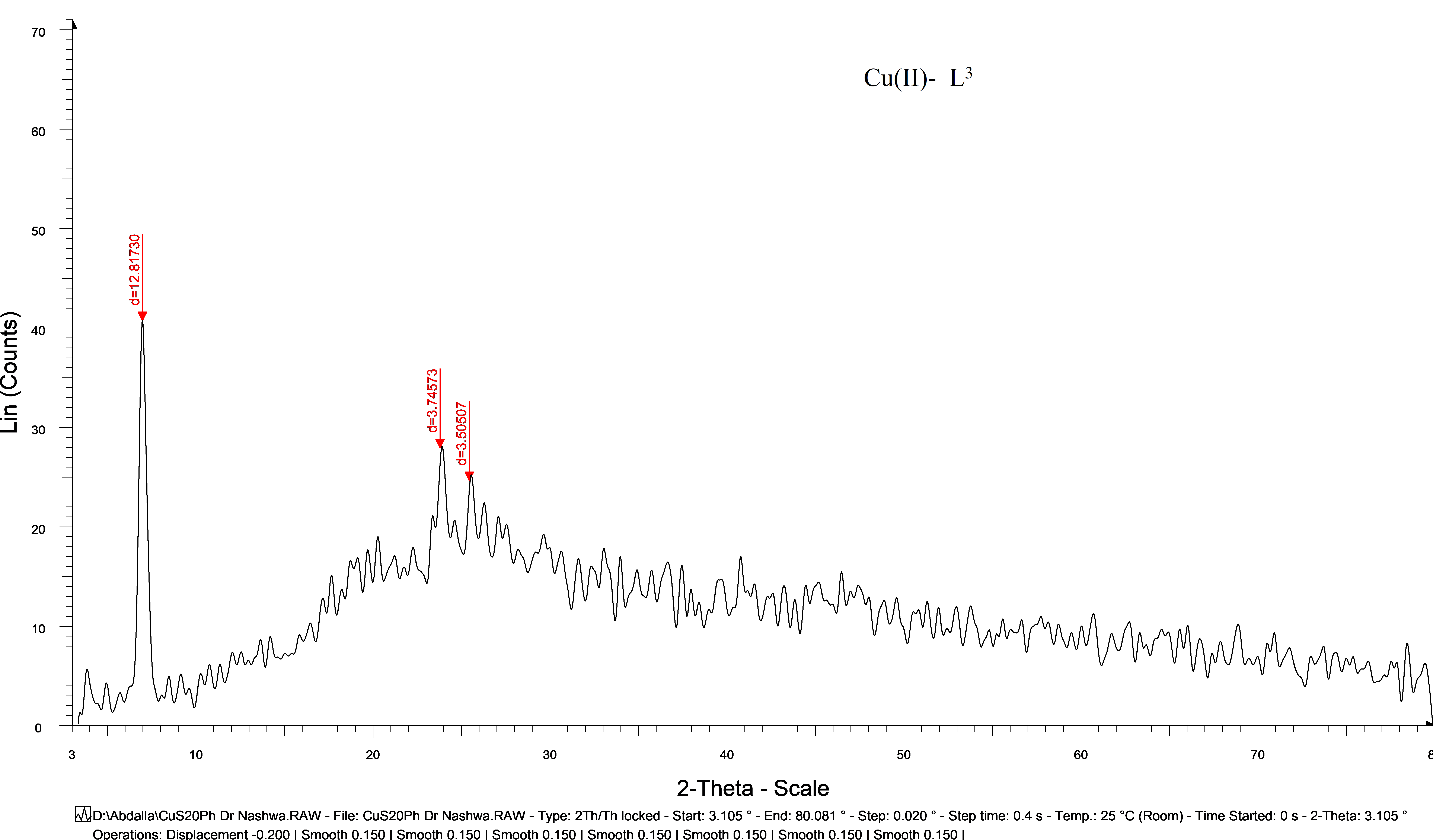 \| \| --- \| \| 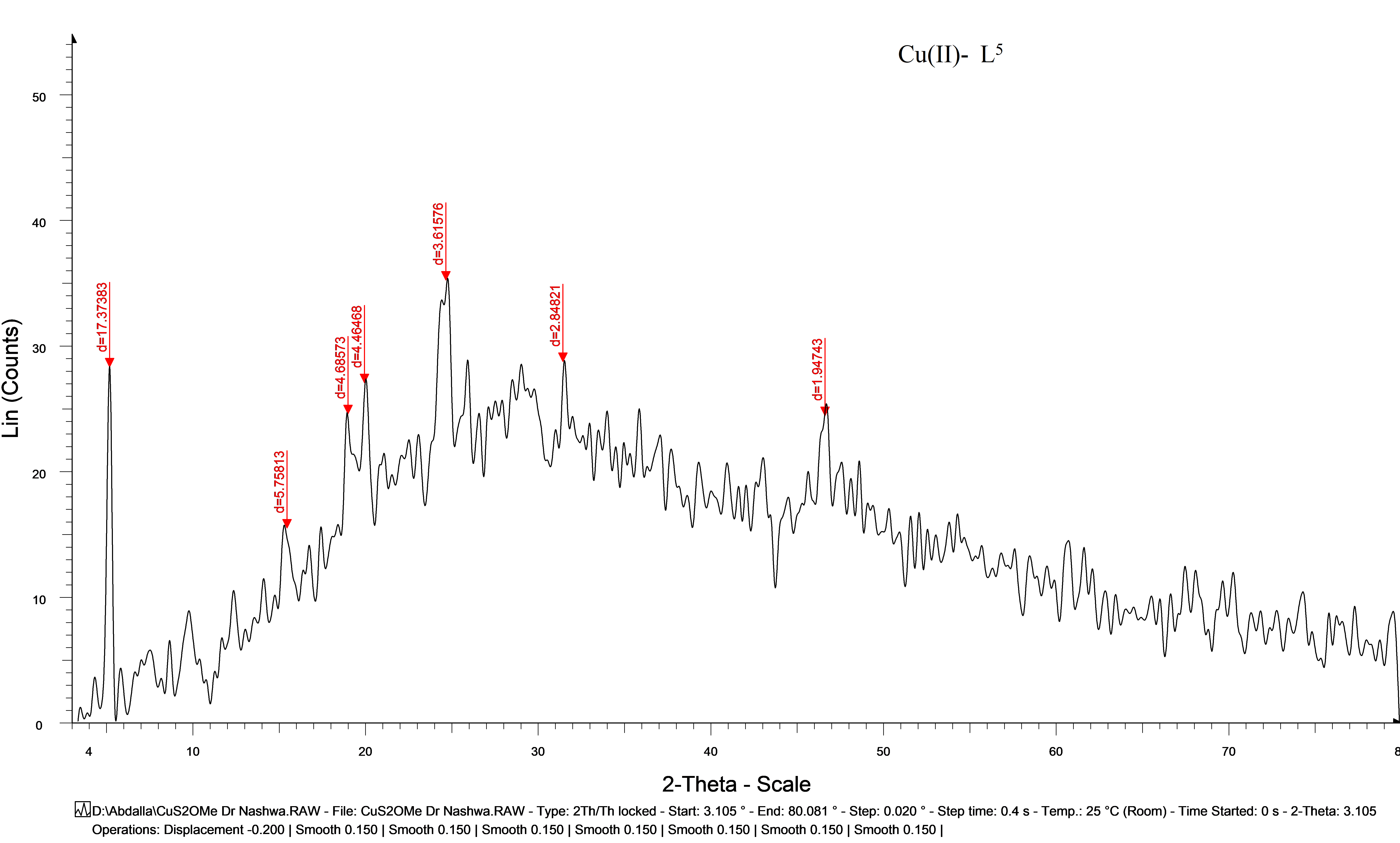 \| \| **Figure S6.** XRD patterns of Cu(II)-L^3^ and Cu(II)-L^5^ complexes \| | | | | | |
| \| 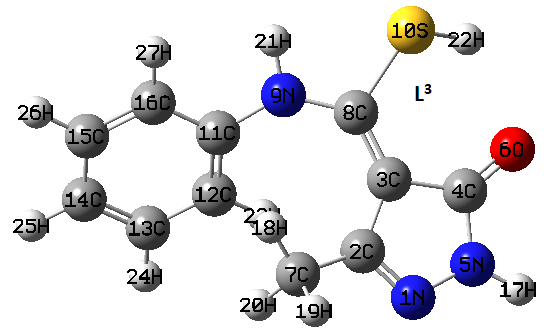 \| 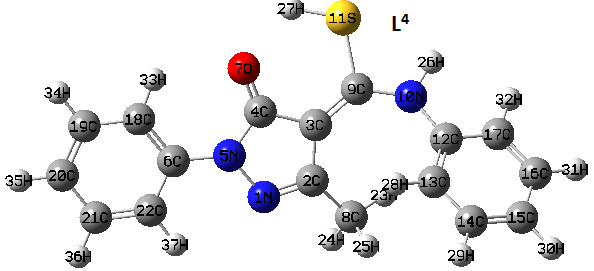 \| \| --- \| --- \| \| 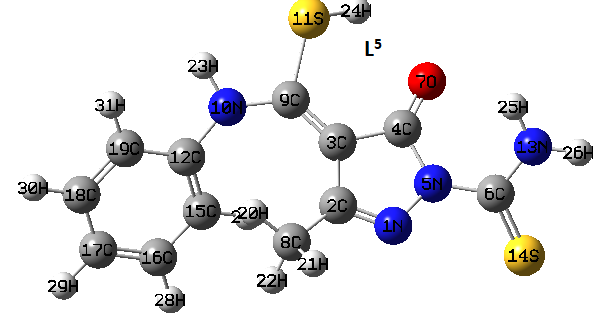 \|  \| \| **Fig. S7.** Optimized structures of pyrazoles derivatives (L^3-5^) \| \| | | | | | |
| 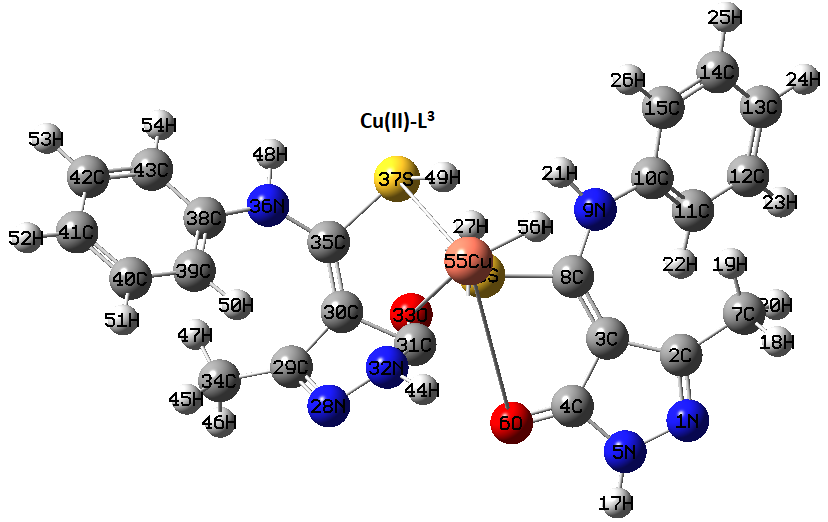 | 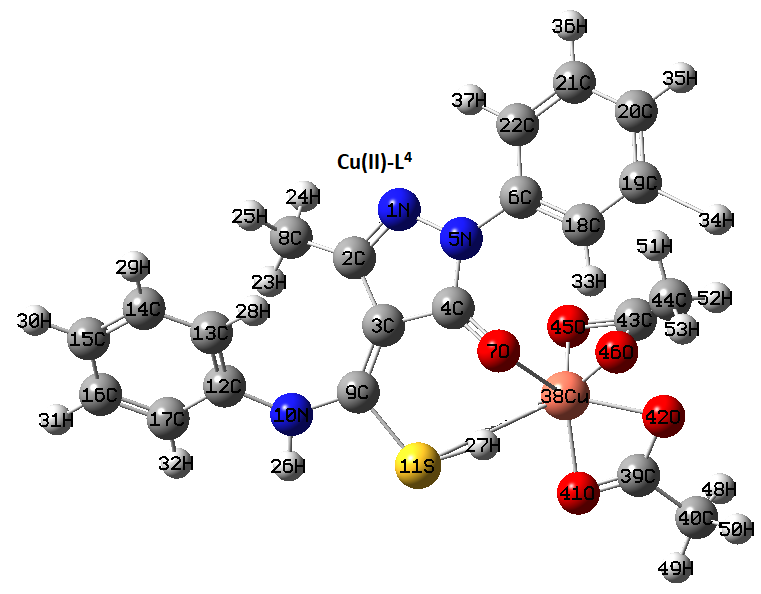 | |  |  |  |
| 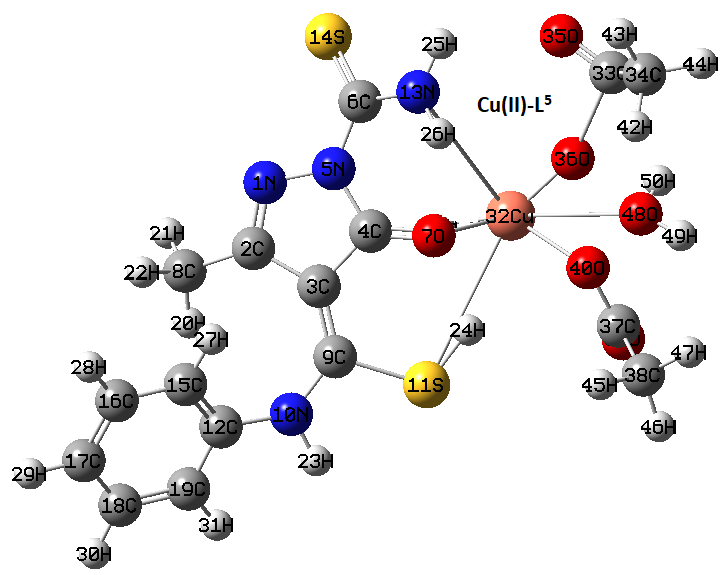 |  | |  |  |  |
| **Figure S8** Optimized structures of new Cu(II)-L^3^ , Cu(II)-L^4^ and Cu(II)-L^5^ complexes | | |  |  |  |

|  |
| --- |
| **Figure S9.** Mulliken atomic charges for pyrazole derivatives (L^3-5^) |

| 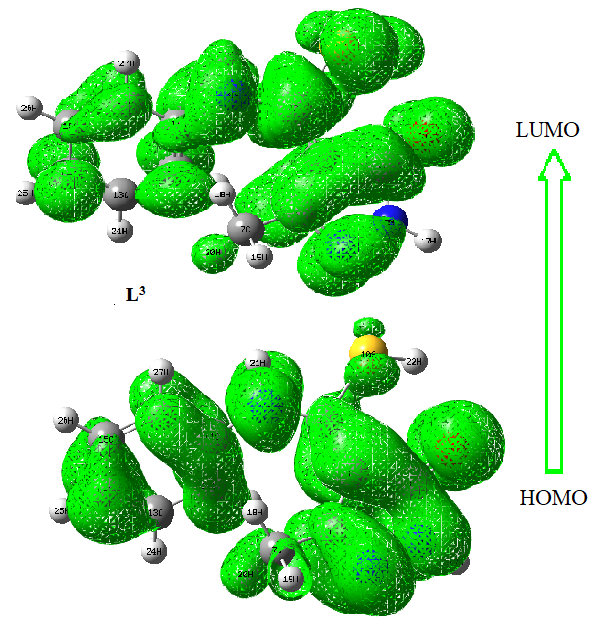 | 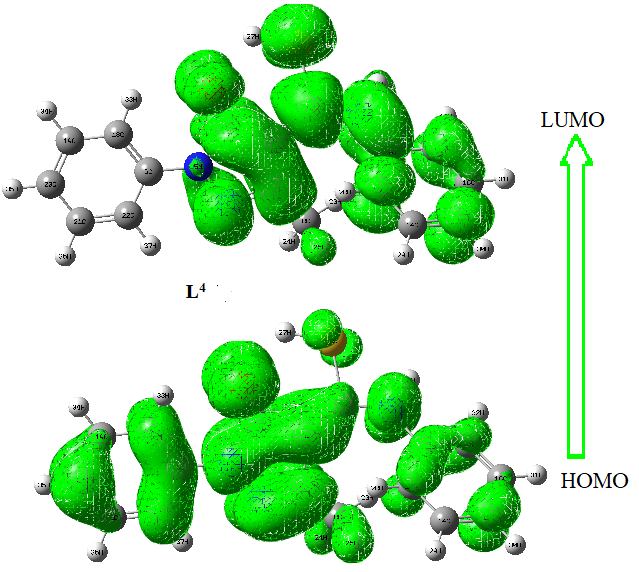 |
| --- | --- |
| 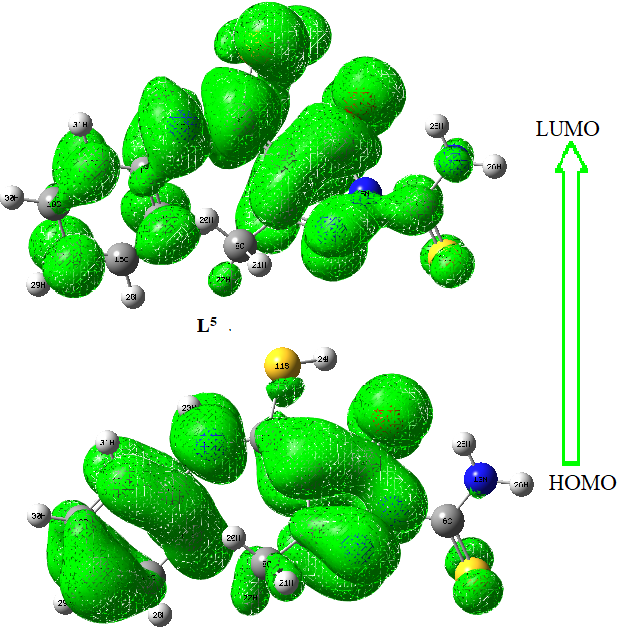 | |
| **Figure S10.** HOMO and LUMO levels of pyrazole derivatives (L^3-5^) | |

| 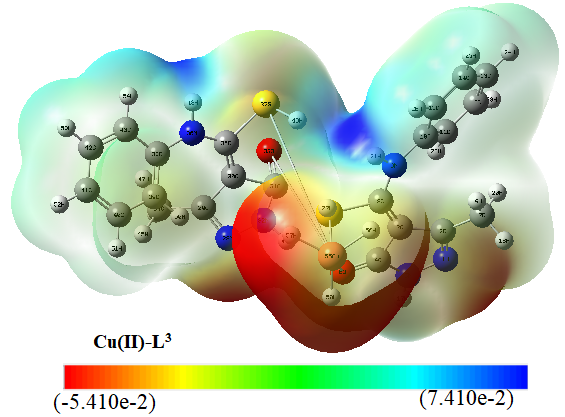 | 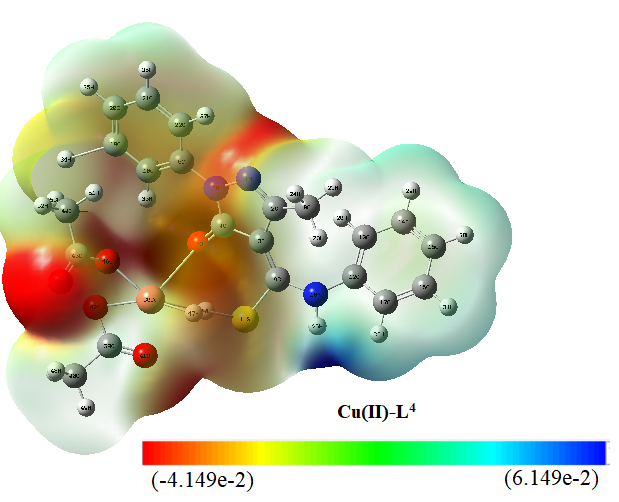 |
| --- | --- |
| 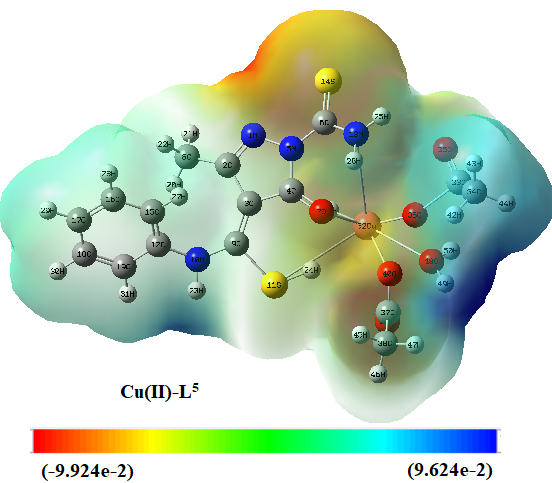 |  |
| **Figure S**11. MEP maps of new Cu(II)-L^3^ , Cu(II)-L^4^ and Cu(II)-L^5^ complexes | |

| 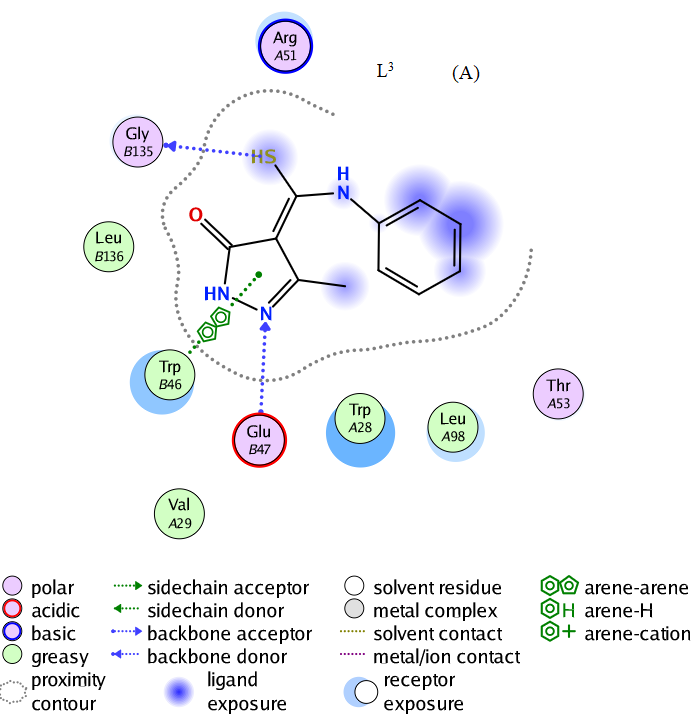 | 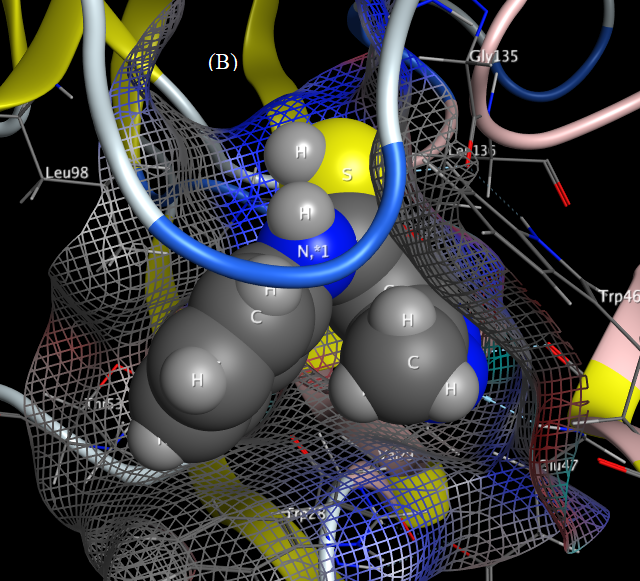 |
| --- | --- |
| 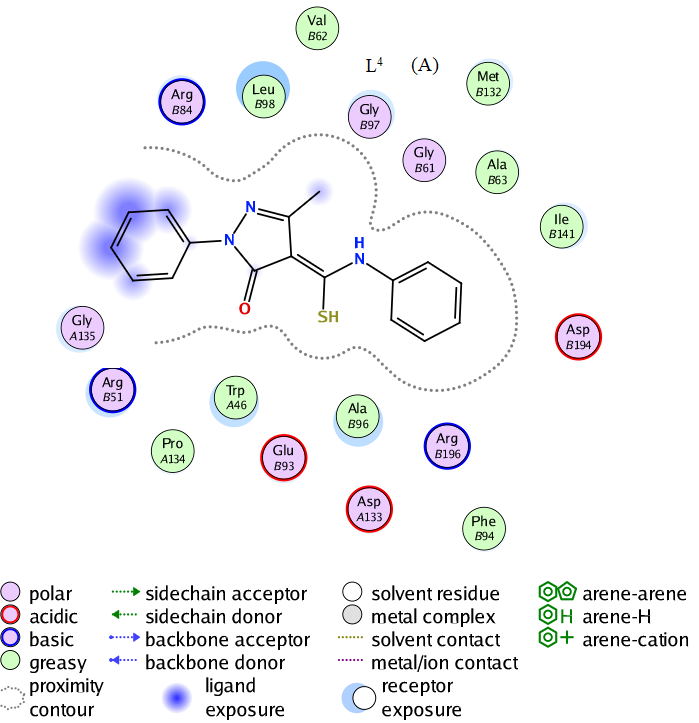 | 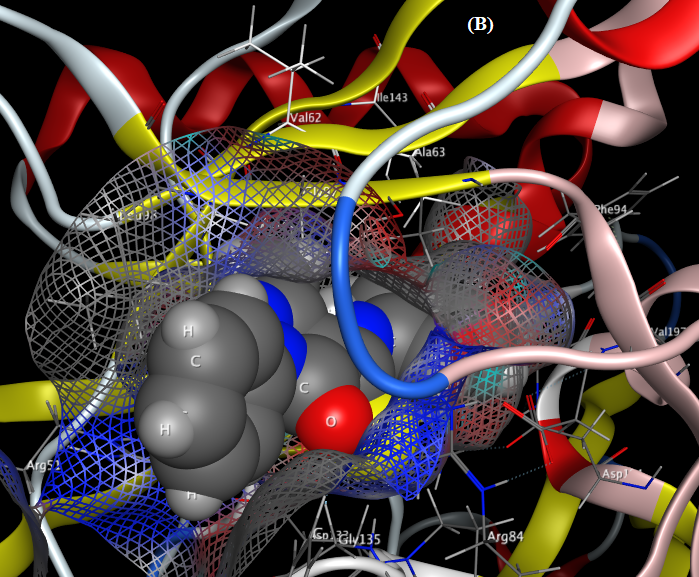 |
| 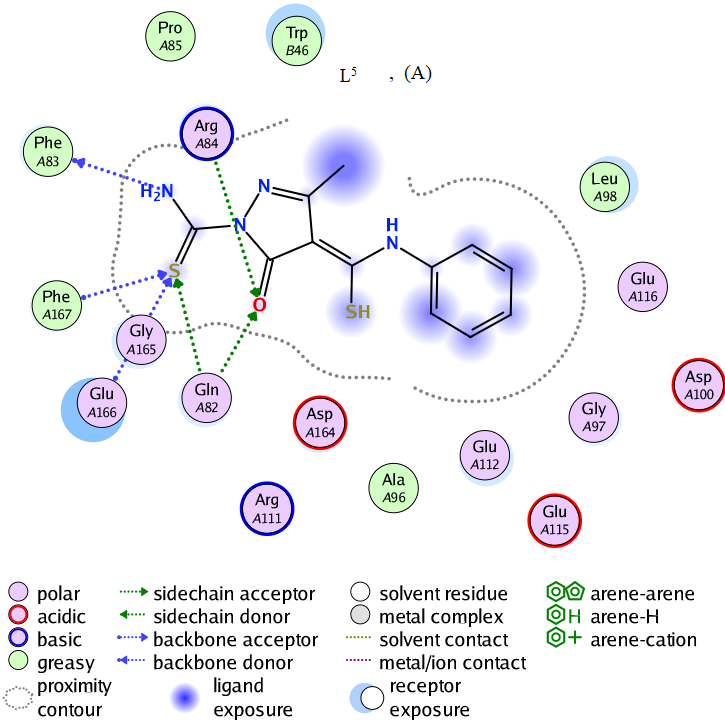 | 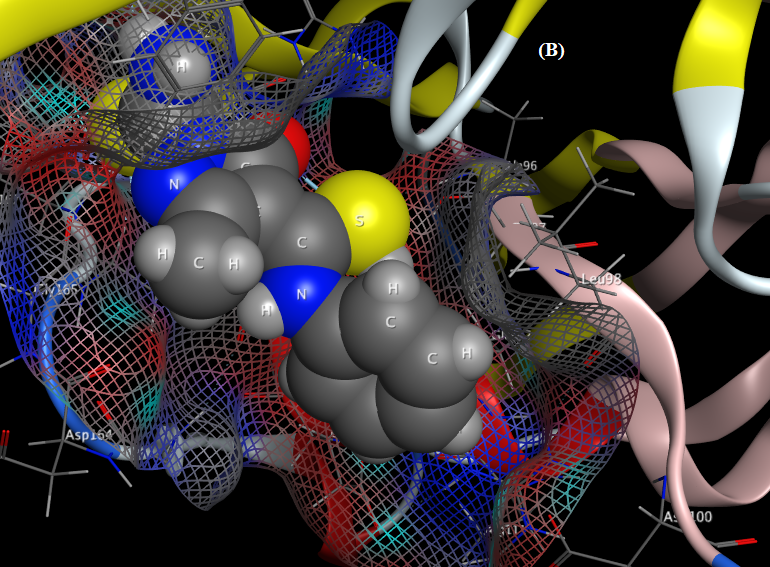 |
| **Figure S12.** Docking validity (A) and electrostatic surface (B) maps of pyrazoles derivatives (L^3-5^) versus 6gru protein | |

**Table S1**. Thermogarvimetric degradation pathway for Cu(II)-pyrazole complexes

| **Complexes** | **Steps** | **T. range (°C)** | **Decomposed** | **Weight loss; Calcd (Found %)** |
| --- | --- | --- | --- | --- |
| 1)[Cu(L^3^)_2_](OAc)_2_ | 1^st^  2^ed^ 3^rd^  residue | 191.34-270.91 271.34-501.21 501.21-703.27 | -(OAc)+C_11_H_11_N_3_S -(OAc)+C_6_H_5_ -C_2_H_6_N_3_OS CuO+3C | 42.63(42.73) 21.00(21.06) 18.54(18.44) 17.83(17.77) |
| 2)[Cu(OAc)_2_(L^4^)](H_2_O) | 1^st^  2^ed^ 3^rd^  4^th^ residue | 41.34-103.82 103.82-367.63 367.64-385.71 385.71-525.91 | -H_2_O+OAc+CH_4_ -OAc -C_6_H_5_ -C_8_H_6_N_3_S CuO+2C | 18.29(18.31) 11.60(11.61) 15.13(15.01) 34.62(34.60) 20.35(20.47) |
| 3)[Cu(OAc)(L^5^)(H_2_O)](OAc)(H_2_O) | 1^st^  2^ed^ 3^rd^  4^th^ residue | 54.63-215.21 215.22-300.31 300.31-450.11 450.11-552.34 | -H_2_O+H_2_S -OAc -OAc+CH_4_ -C_6_H_6_N+H_2_S+N_2_+NO CuO+5C | 10.21(10.25) 11.58(11.50) 14.72(14.74) 36.12(36.12) 27.37(27.39) |

**Table S2.** Estimated physical parameters (ev) under optimal RTD-B3LYP-FC condition in ethanol

| **Compound** | **E_H_** | **E_L_** | **E_H_ -E_L_** | **E_l_-E_h_** | ***x*** | **µ** | **η** | **S(eV-1)** | **ω** | **ϭ** | **D_(Debye)_** | **E_(A.U.)_** |
| --- | --- | --- | --- | --- | --- | --- | --- | --- | --- | --- | --- | --- |
| 1)L^3^ | -0.2120 | -0.0725 | -0.1395 | 0.1395 | 0.1422 | -0.1423 | 0.0697 | 0.0349 | 0.1451 | 14.3400 | 5.8195 | -1063.2043 |
| 2)Cu(II)-L^3^ | -0.1911 | -0.1722 | -0.0189 | 0.0189 | 0.1817 | -0.1817 | 0.0100 | 0.0047 | 1.7424 | 105.5966 | 10.4331 | -3767.8959 |
| 3)L^4^ | -0.2096 | -0.0730 | -0.1366 | 0.1366 | 0.1413 | -0.1413 | 0.0683 | 0.0341 | 0.1462 | 14.6424 | 6.8820 | -1294.2036 |
| 4)Cu(II)-L^4^ | -0.2078 | -0.1603 | -0.0476 | 0.0476 | 0.1841 | -0.1841 | 0.0238 | 0.0119 | 0.7125 | 42.0610 | 6.9062 | -3391.5343 |
| 5)L^5^ | -0.2220 | -0.0824 | -0.1396 | 0.1396 | 0.1522 | -0.1522 | 0.0698 | 0.0349 | 0.1659 | 14.3266 | 9.7749 | -1554.7972 |
| 6)Cu(II)-L^5^ | -0.2042 | -0.1507 | -0.0535 | 0.0535 | 0.1774 | -0.1774 | 0.0268 | 0.0134 | 0.5881 | 37.3622 | 13.0671 | -3728.6061 |

**Table 3S.** QSAR parameters for pyrazoles and their Cu(II) complexes

| **The parameters** | **L^3^** | **Cu(II)-L^3^** | **L^4^** | **Cu(II)-L^4^** | **L^5^** | **Cu(II)-L^5^** |
| --- | --- | --- | --- | --- | --- | --- |
| **Surface area (grid) (Å)** | 406.10 | 692.97 | 518.12 | 650.92 | 470.97 | 608.79 |
| **Volume(Å)** | 649.68 | 1227.29 | 859.90 | 1154.16 | 770.21 | 1074.34 |
| **Hydration energy(k cal/mol)** | -9.64 | -16.80 | -6.56 | -9.78 | -11.94 | -15.91 |
| **Log p** | 0.93 | 0.34 | 1.67 | 0.96 | 1.48 | 0.01 |
| **Reactivity(Å)** | 69.98 | 141.83 | 99.46 | 123.43 | 86.33 | 109.28 |
| **Polarizability(Å)** | 25.36 | 44.80 | 35.02 | 41.45 | 31.80 | 38.03 |
